# Channel-specific differential effects of bacterial mechanosensitive channels for ultrasound neuromodulation in precision sonogenetics

**DOI:** 10.7150/thno.119770

**Published:** 2026-01-01

**Authors:** Xin Li, Chenguang Zheng, Yutao Tian, Dong Ming

**Affiliations:** 1 Academy of Medical Engineering and Translational Medicine, Medical College, Tianjin University, Tianjin, China.; 2 Tianjin Key Laboratory of Brain Science and Neuroengineering, Tianjin, China.; 3 Haihe Laboratory of Brain-Computer Interaction and Human-Machine Integration, Tianjin, China.

**Keywords:** mechanosensitive ion channel, ultrasound stimulation, hippocampus, local field potential, sonogenetics

## Abstract

**Rationale:** Ultrasound neuromodulation offers promising therapeutic potential, but its effectiveness is limited by imprecise targeting of neural circuits. Engineering mechanosensitive ion channels can enhance ultrasound sensitivity, providing a more precise approach for targeted neuromodulation. This study aimed to compare three bacterial mechanosensitive channels (MscL-G22S, MscL-G22N, and MscS) for mediating ultrasound-responsive hippocampal activity to identify optimal candidates for precision sonogenetics applications.

**Methods:** We expressed MscL-G22S, MscL-G22N, and MscS in the rat hippocampus using AAV vectors and applied focused ultrasound stimulation at various intensities while recording local field potentials. Neural oscillatory patterns, ultrasound-evoked potentials, behavioral outcomes, immunohistology, and transcriptomic analyses were conducted to assess response consistency, efficacy, and biosafety.

**Results:** Each channel conferred distinct neuromodulatory signatures: MscL-G22S exhibited remarkable ultrasound sensitivity with non-monotonic intensity-response amplification of evoked potentials (2.3-fold increase at maximum intensity), and accelerated response timing (latency reduction). Notably, MscL-G22N showed weaker ultrasound responses despite having a lower mechanical threshold than G22S, suggesting ultrasound sensitivity depends on factors beyond mechanical gating thresholds. Conversely, MscS displayed diminished responses at higher intensities. No statistically significant differences were detected in behavior assessments and histology evaluations. All channels maintained normal anxiety indices, spatial memory, and neuronal morphology, though MscS selectively increased depressive-like behaviors. Transcriptomic analysis revealed that MscS demonstrated exceptional genomic compatibility with minimal off-target gene alterations (9 vs. >400 in MscL variants).

**Conclusion:** This characterization provides insights for potential precision sonogenetics applications: MscS offers a biosafety-optimized option with minimal genomic footprint, whereas MscL-G22S enables modulation of neural oscillations. These findings contribute to the development of customized neuromodulation approaches for targeting pathological circuits in neurological disorders.

## Introduction

Non-invasive neuromodulation technologies have transformed both basic neuroscience research and clinical applications by enabling reversible neural circuit manipulation with minimal tissue damage 1-3. Ultrasound-based neuromodulation offers unique advantages for both research and therapeutic applications, permitting deep brain stimulation with focal precision of approximately 1 to 2 mm 4, 5. This superior spatial resolution positions ultrasound as a promising modality for treating disorders affecting deep brain structures inaccessible to other non-invasive techniques. Ultrasound neuromodulation operates through acoustic radiation force 6, 7, cavitation effect 8, and thermal gradients 9, 10. Research has established that acoustic radiation force primarily acts by activating mechanosensitive ion channels embedded in neuronal membranes, creating a direct mechanotransduction pathway with therapeutic potential 11-13. Various mechanosensitive channels respond to ultrasound stimulation in both *in vitro* and *in vivo* studies, including mammalian channels such as the Piezo family 14-17, the K_2_P family 18-21, the TRP family 22-28, mPrestin 29, as well as bacterial channels like MscL 30-33, each offering distinct biophysical properties and activation thresholds with implications for targeted applications.

Engineering of these mechanosensitive channels has shown considerable promise for enhancing ultrasound sensitivity beyond their native capabilities, potentially enabling more precise control of neural activity. The bacterial channels, due to their unique biophysical properties, have developed into a novel tool for ultrasound-mediated neuromodulation. Studies with the bacterial MscL mutant (I92L, G22S) expressed in cultured neurons demonstrated that ultrasound could induce action potentials at pressures as low as 0.25 MPa 34 and 0.15 MPa 30, significantly improving sensitivity compared to native channels. Especially, the G22 residue due to its pivotal role in the channel's constriction site, enabling systematic tuning of mechanical thresholds 35, 36. Subsequent studies confirmed that low-intensity ultrasound effectively activates MscL-G22S, increasing intraneuronal calcium levels 30, 31. *In vivo* studies demonstrate that ultrasound stimulation induces electromyographic responses and elevates c-Fos expression, confirming neural activation 30, 31, 37.

The application of engineered mechanosensitive channels also shows significant therapeutic potential in animal models of neurological disorders, including epilepsy 33, Parkinson's disease 31, 38, Alzheimer's disease 39, and visual restoration 32. These conditions represent a substantial clinical burden globally, with current pharmacological interventions often limited by significant side effects or insufficient efficacy in treatment-resistant cases. The capacity to target aberrant oscillatory activity—a pathophysiological hallmark of multiple neurological conditions—represents a promising avenue for therapeutic intervention that could overcome the limitations of current treatments. Mechanosensitive channels enable the modulation of specific neural circuits with temporal and spatial precision unattainable with conventional pharmacology or less focal neuromodulation approaches. Despite this promise, a critical knowledge gap exists in our understanding of ultrasound-evoked electrophysiological responses mediated by different mechanosensitive channels 40. We lack comparative data on their efficacy, temporal response characteristics, and frequency-specific effects on neural activity—information essential for the rational design of ultrasound-based neuromodulation paradigms for specific clinical applications. This knowledge gap significantly hinders further refinement and precision of ultrasound-based neuromodulation as a therapeutic modality.

To address this knowledge gap, we investigated the electrophysiological characteristics of ultrasound-mediated neuromodulation through three bacterial mechanosensitive channels: MscL-G22S, MscL-G22N, and MscS. Specifically, we compared the established MscL-G22S benchmark with MscL-G22N—an intra-family variant at the same G22 residue—and MscS, an inter-family channel with a distinct structure, to assess the specificity and mechanistic diversity of sonogenetic activation. Local field potentials (LFPs) were recorded from the rat hippocampus, a region that is not only critical for learning and memory with well-characterized oscillatory patterns, but its relevance to cognitive disorders—including Alzheimer's disease, temporal lobe epilepsy, and post-traumatic stress disorder—also makes it a clinically significant target for testing neuromodulation approaches. We analyzed ultrasound-evoked potentials and changes in oscillatory patterns across frequency bands, focusing on peak amplitude and latency as key indicators of neural response timing and strength. These parameters are directly relevant to clinical applications requiring precise temporal control of neural activity and have implications for probing functional connectivity patterns in both healthy and diseased brain states.

Our findings demonstrate that mechanosensitive channels mediate ultrasound stimulation with distinct and predictable electrophysiological signatures, offering unprecedented opportunities for targeted neuromodulation with potential therapeutic applications. We observed channel-specific differences in ultrasound sensitivity, neural response characteristics, and frequency-band modulation that provide a foundation for developing more precise neuromodulation approaches. Importantly, we discovered that ultrasound neuromodulation efficacy does not correlate directly with established mechanical gating thresholds, suggesting additional biophysical factors influence channel responsiveness to ultrasonic stimulation. These insights will guide the future engineering of optimized sonogenetic tools for targeted intervention in specific neural circuits, potentially addressing treatment-resistant neurological and psychiatric disorders through non-invasive, spatially-precise neuromodulation.

## Materials and Methods

### Animals and experimental setting

**Figure 1:**
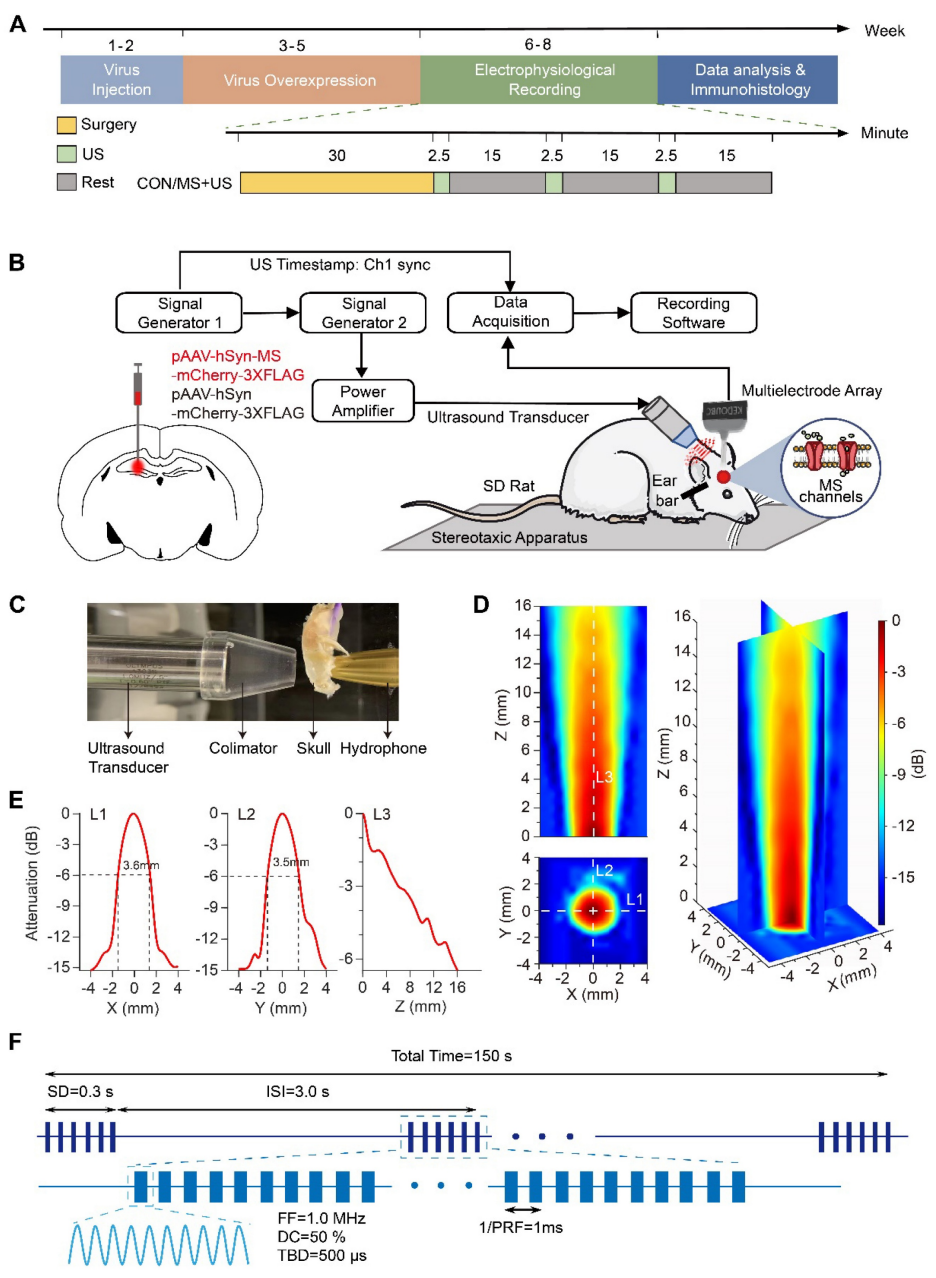
** Diagrams of the experimental setup and ultrasound stimulation schematic. (A)** Experimental procedure timeline. US, ultrasound stimulation; MS, mechanosensitive.** (B)** Integrated ultrasound stimulation and LFP recording system setup. **(C)** Ultrasound transducer calibration with a 3D-printed acoustic collimator and a rat skull. **(D)** 1.0 MHz focused ultrasound transducer's acoustic field distribution in X-Y and X-Z planes in the open field. **(E)** Sound attenuation profile along the white dotted lines (L1, L2, L3) shown in (D). **(F)** Ultrasound stimulation parameters schematic. SD, sonication duration; ISI, inter-stimulus interval; FF, fundamental frequency; DC, duty cycle; TBD, tone burst duration; PRF, pulse repetition frequency.

Forty male Sprague-Dawley rats (Beijing Vital River Laboratory Animal Technology Co., Ltd.), aged approximately 8 weeks and weighing between 250-300 g, were used in this study. Rats were housed in pairs under a 12-hour light/dark cycle (light: 8:00 pm to 8:00 am; dark: 8:00 am to 8:00 pm) in a temperature- and humidity-controlled environment (25 ± 1 °C, 60%-65% humidity) with access to food and water ad libitum. All procedures were approved by the Animal Care and Use Committee of Tianjin University. The experiment setting and procedures as shown in Figure 1A. The group settings as “CON” (control, sham-injected with an empty viral vector and subjected to identical surgical and behavioral protocols as experimental groups), “MscL-G22S” (large conductance mechanosensitive channel, G22S mutant), “MscL-G22N” (large conductance mechanosensitive channel, G22N mutant), and “MscS” (small conductance mechanosensitive channel).

### Virus packaging and stereotaxic injection

High-titer adeno-associated viral vectors (AAV) were obtained from commercial sources (OBiO Technology, Shanghai, China), packaged, and stored at -80 °C until use. The sequences for MscL-G22S, MscL-G22N, and MscS were fused with the fluorescent reporter to enable visualization of expression. Viral information is shown in Supplementary Table S1.

Animals were anesthetized with 5% isoflurane in 1 L/min O₂ and maintained under 2%-3% isoflurane during surgery. The body temperature was maintained and monitored throughout the whole surgical procedure. Craniotomies were performed, and the virus was microinjected into the hippocampus (AP -3.8 mm, ML 2 mm, DV 2.6 mm) using stereotaxic techniques. Viruses were delivered at 0.1 μl/min, and the micro-syringe was held in place for 10 minutes to stabilize the injection. After the retraction of the pipette, a 5-minute pause was taken before completely withdrawing the syringe. The puncture site was disinfected and sutured, and rats were returned to their home cages for 4 weeks to allow for viral overexpression in the targeted region.

### Ultrasound stimulation system and calibration parameters

The ultrasound system (Figure 1B) comprised two function generators (DG4162 and DG822; RIGOL, China) for waveform generation, an RF amplifier (SWA400B; North Star, China) for signal amplification, a 1.0 MHz immersion transducer (V303; Olympus, Japan) for ultrasound generation, and a 3D printed acoustic collimator for beam focusing. Ultrasound pressure calibration was conducted using a calibrated hydrophone (HGL0800; ONDA, USA) to quantify transcranial attenuation through fresh rat skulls (Figure 1C), ensuring precise pressure delivery to the targeted brain regions. The spatial distribution of ultrasound pressure was mapped in degassed water using 0.2 mm step sizes, and focal area diameters were measured (Figure 1D,1E). The ultrasound parameters were optimized for mechanosensitive ion channel activation (Figure 1F), fundamental frequency (FF) of 1.0 MHz, a pulse repetition frequency (PRF) of 1.0 kHz, a duty cycle (DC) of 50%, a 0.5 ms tone burst duration (TBD), a 0.3 s stimulation duration (SD), and inter-stimulation intervals (ISI) of 3 s. Ultrasound intensities were calibrated to spatial peak temporal average intensities (I_spta_) and peak negative pressure (PNP) of 100 mW/cm² (0.15 MPa), 250 mW/cm² (0.25 MPa), and 400 mW/cm² (0.35 MPa), spanning the activation threshold range of the engineered mechanosensitive ion channels. Our maximum parameters (PNP = 0.35 MPa, I_spta_ = 400 mW/cm², I_sppa_ = 800 mW/cm²) delivered at a center frequency of 1.0 MHz resulted in a maximum mechanical index (MI) of 0.35 (Supplementary Table S2), which is substantially below the FDA's safety limit for inertial cavitation (MI = 1.9 for general tissue). Based on this low MI, the risk of cavitation was considered negligible, and thus active cavitation monitoring was not performed. The temperature elevation was estimated according to standard bio-heat transfer equations under continuous exposure conditions (Supplementary Table S3, Figure S3). The measured maximum temperature rise after 50 pulses (Supplementary Table S4, Figure S4) was below 0.2 °C across all intensities tested. The initial stimulation intensity (100 mW/cm²) was selected based on established literature 4, 41, representing the lower threshold for low-intensity focused ultrasound without tissue damage. The setup enabled precise targeting of the hippocampus at a 45 ° angle, optimizing ultrasound wave propagation through the skull while minimizing standing wave artifacts.

### Craniotomy surgery and electrode positioning

Four weeks after viral overexpression in the hippocampus, rats were anesthetized with 3% isoflurane in O₂ for craniotomy surgery and maintained with 1.0% isoflurane during electrophysiological recordings. Body temperature was regulated at 37-38°C using a feedback-controlled heating pad with rectal temperature monitoring. Rats were secured in a stereotaxic frame (RWD Life Technology, China), and a midline incision was made to expose the skull. The bregma and lambda were aligned to the horizontal plane of the skull. A cranial window (AP: -3.0 to -4.6 mm, ML: 1.2 to 2.8 mm, relative to bregma), which corresponds to regions with confirmed viral expression, was carefully created using a high-speed stereotaxic drill under stereomicroscopic guidance, with intermittent saline cooling to prevent heat-induced tissue damage. The exposed cortical surface was continuously maintained with sterile 0.9% physiological saline to prevent desiccation and minimize cortical inflammation.

### *In vivo* electrophysiology recording

To minimize electromagnetic interference, all *in vivo* recordings were performed inside a shielded Faraday cage. The ultrasound-generating system was positioned outside the cage, and all recording and stimulation equipment was connected to a common ground. A dedicated timestamp/sync channel marked ultrasound onset for alignment of signal analysis windows. Electrophysiological recordings were conducted using Epoxylite-coated tungsten microelectrodes with a resistance of 1 MΩ. A 16-channel tungsten array (4x4, 5 mm length, 50 μm tip diameter, 100 μm spacing; KedouBC Technology, Suzhou, China) was interfaced with the Intan RHS2000 acquisition system (Intan Technologies, USA) with appropriate impedance matching. Broadband neural signals (0.5 Hz to 7.5 kHz) were digitized at a 20 kHz sampling rate.

### Electrophysiology data analysis

Data analysis was performed using custom MATLAB 2020b code (The MathWorks). Raw data were converted into a MATLAB-compatible format. To mitigate electrical noise, differential recording with a grounded reference electrode to minimize common-mode noise, notch filters to remove the 50 Hz power-line interference, and its specific odd harmonics (e.g., 150, 250, 350 Hz) were applied. LFPs were extracted using bandpass filtering from 0.5 to 100 Hz (Butterworth filter order 2), and baseline correction was performed on the filtered data. Power spectral densities were estimated using the Welch algorithm to compute total power and power within conventional frequency bands. Relative power (RP) for each frequency band (delta: 0.5~4 Hz, theta: 4~8 Hz, alpha: 8~12 Hz, beta: 12~30 Hz, gamma: 30~100 Hz) was calculated by using a Hanning window to avoid the power spectral density (PSD) leakage and normalizing to the total absolute power in the 0.5-100 Hz range. To remove the ultrasound artifact, we conducted post-mortem control (Supplementary Figure S1) and sham control experiments (Supplementary Figure S2), after filtering any signals matching the non-biological artifact waveforms (identified from our post-mortem control) were flagged and discarded. Then the cleaned signal underwent baseline correction for further analysis. Ultrasound evoked potentials (UEPs) were analyzed within a 3s window (-1 to 2 s relative to ultrasound onset), with peak amplitude and latency measured from 0 s to 0.5 s to quantify the direct neural responses to mechanosensitive channel activation. Latency is defined as the time to the first negative deflection (N1) within 0-500 ms after ultrasound onset; peak amplitude refers to the N1 amplitude.

### Behavioral assessment of mechanosensitive channels expressed animals

Twenty rats were divided into four experimental groups. After four weeks of virus expression, a series of behavioral assessments was conducted, including (1) locomotor activity and anxiety (open-field test, OFT; elevated plus maze, EPM); (2) spatial working memory (Y-maze novel arm, YNA; Y-maze spontaneous alternation, YSA); and (3) depression-like behavior (forced swim test, FST). In the OFT 42, rats were placed at the center of a 100 × 100 cm chamber with 40 cm high walls under controlled lighting conditions. Behavior was recorded for 10 minutes, measuring time spent and entries into the center zone as indices of anxiety-like behavior. In the EPM 43, rats were placed on the central platform of a plus-shaped maze with two open and two closed arms. During the 10-minute test, time spent, distance traveled, and entries into the open arms were quantified as inverse measures of anxiety. The YNA test 44, 45 consisted of a 10-minute sample phase with access to only two arms, followed by a 5-minute test phase after a two-hour delay, during which the previously closed "novel arm" was accessible. For the YSA test 46, rats were allowed free exploration of all three arms. Alternation behavior (defined as consecutive entries into all three arms without repetition) was recorded and expressed as a percentage of the maximum possible alternations [(number of alternations) / (total arm entries-2) ×100]. For the FST 47, 48, rats were placed individually in transparent cylinders filled with 25°C water to a depth of 30 cm for 6 minutes. Immobility time during the last 4 minutes was quantified as an index of behavioral despair. All behavioral tests were video-recorded and analyzed using SMART 3.0 tracking software (Panlab Harvard Apparatus, Spain) with standardized detection settings optimized for rat size and contrast.

### Histological verification and immunohistochemical analysis

Following the completion of baseline behavioral experiments, histological sections were prepared to verify the potential impact of channel overexpression on tissue morphology and inflammatory responses. Rats were euthanized with a lethal dose of urethane (2.0 g/kg, intraperitoneally), followed by transcardial perfusion with ice-cold phosphate-buffered saline (PBS, 200 mL) and 4% paraformaldehyde (PFA, 250 mL at 10 mL/min) for tissue fixation. The brains were extracted, post-fixed in 4% PFA for 24 hours at 4°C, then cryoprotected through ascending sucrose gradients (15% for 24h, 30% until sinking). Brain tissue was embedded in an optimal cutting temperature compound and sectioned coronally at 30 μm thickness using a freezing microtome (CM1950, Leica, Germany). For expression analysis, immunofluorescence was used to identify the nuclei using DAPI sealed tablets (Beyotime, China), the reporter protein was used to mark the target mechanosensitive channels, and the quantification of expression level, the mean intensity, and the percentage of positive cells were calculated by using ImageJ software. For morphological analysis, sections were stained with hematoxylin and eosin to assess neuronal integrity. For microglial activation assessment, Iba1 immunostaining was performed using rabbit polyclonal anti-Iba1 primary antibody (1:500, FUJIFILM Wako, Japan) and visualized with either Alexa Fluor 488 goat anti-rabbit IgG or Alexa Fluor 555 donkey anti-rabbit IgG secondary antibodies (both 1:500, Beyotime, China) for double-labeling experiments. Nuclei were counterstained with DAPI (Beyotime, China) for 10 minutes at room temperature. Fluorescent images were acquired using a confocal laser-scanning microscope (A1R, Nikon, Japan), standardized laser power, and gain settings optimized for each fluorophore. The Iba1+ cells were counted manually by an investigator blinded to the experimental conditions, using ImageJ software. A consistent counting frame was applied to each field of view to ensure unbiased counting. Cell counts were normalized and expressed as the number of cells per graph. All statistical analyses for cell counting were performed using GraphPad Prism 9.5. Data normality was assessed using the Shapiro-Wilk test. Depending on normality and homogeneity of variance, comparisons between groups were conducted using a Kruskal-Wallis test followed by Dunn's post-hoc comparisons for non-normally distributed data.

### RNA sequencing and analysis

Hippocampal tissues from a subgroup of mechanosensitive ion channels expressed and control rats were dissected when post-behavior tests finished, immediately preserved in RNA stabilization solution, and flash-frozen at -80 °C to ensure optimal RNA integrity. Total RNA was extracted using a TRIzol-based method for transcriptome sequencing (RNA-seq). Gene expression was quantified with feature counts, and differential expression analysis was performed using DESeq2 with significance thresholds of adjusted p-value ≤ 0.05 and absolute log2 fold change ≥ 1. Gene Ontology (GO)was performed to identify enriched biological process (BP), cellular component (CC), and molecular function (MF). GO terms with adjusted p-values ≤ 0.05 were considered significantly enriched 49, 50. We visualized the top 10 significantly enriched GO terms for each category (BP, CC, MF) using enrichment maps and bubble plots to highlight mechanosensitive channel-related molecular signatures.

### Statistical analysis

Data normality was assessed using the Shapiro-Wilk test. Normally distributed data were analyzed using one-way ANOVA followed by Dunnett's multiple comparisons test, while a non-parametric analysis method was used. All group comparisons of electrophysiological measures within a specific frequency band and stimulus intensity were performed using Kruskal-Wallis tests followed by Dunn's multiple-comparison post-hoc tests (two-sided). All data are presented as mean ± standard error of the mean (SEM) unless otherwise specified. No data were excluded from statistical analyses. Statistical significance was defined as *P* < 0.05 for all analyses. Non-significant results are indicated as ns. (*P* > 0.05) in figures and tables. All statistical analyses were performed using GraphPad Prism 9.5 and custom MATLAB scripts.

## Results

### Electrophysiological impact of exogenously expressed mechanosensitive ion channels on hippocampal neural activity

**Figure 2:**
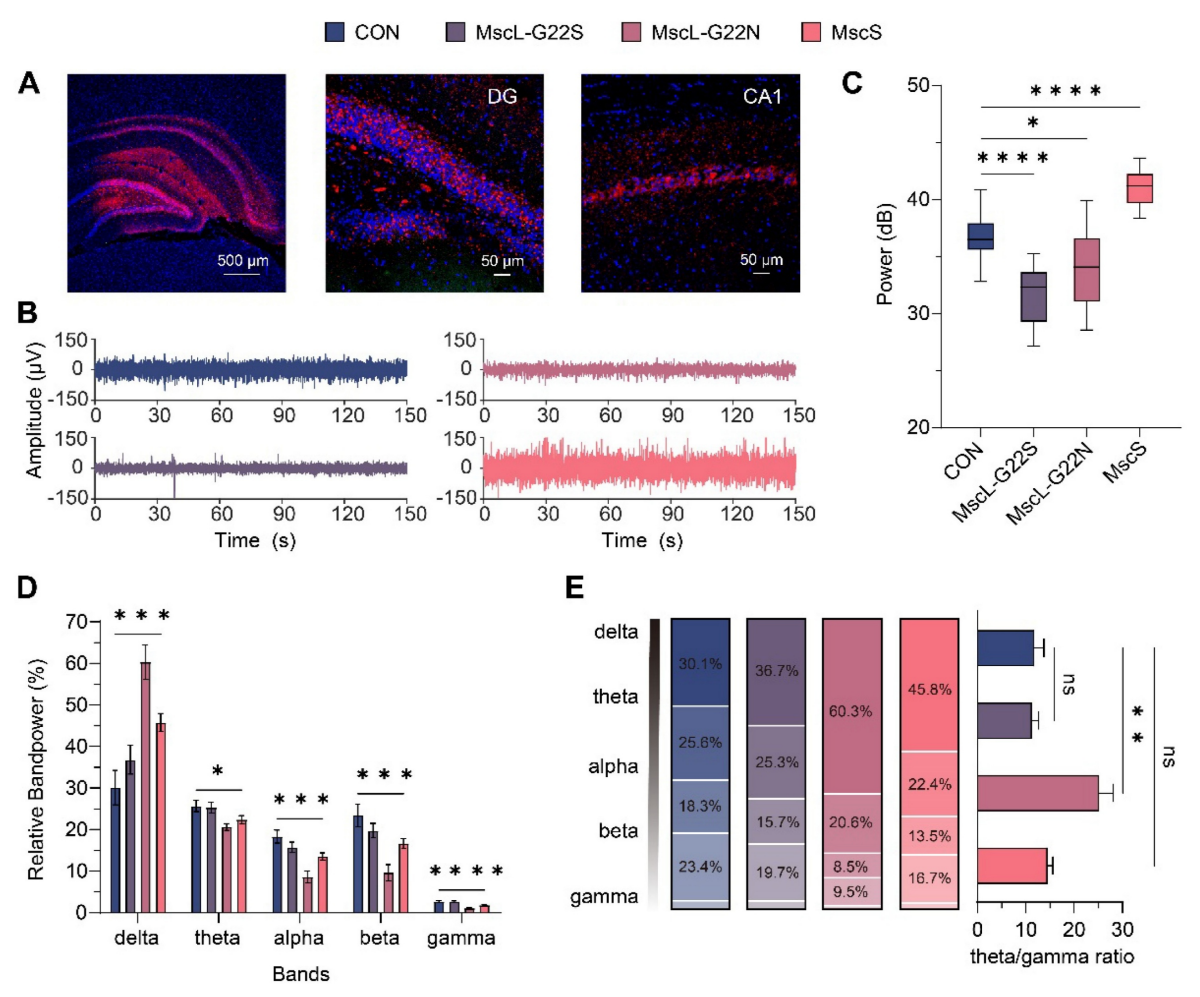
** Spontaneous hippocampal LFP activity with different mechanosensitive ion channels. (A)** Representative fluorescence images of mechanosensitive ion channel expression (mCherry, red; DAPI, blue) in the whole hippocampus (scale bar, 500 μm), DG and CA1 regions (scale bar, 50 μm).** (B)** Comparison of mean waveform amplitudes of spontaneous activities among different mechanosensitive ion channel groups (CON, n=5; MscL-G22S, n=5; MscL-G22N, n=5; MscS, n=5). **(C)** Comparison of the total power of spontaneous neural activities across groups (CON, n=5; MscL-G22S, n=5; MscL-G22N, n=5; MscS, n=5). **(D)** Relative power of different frequency bands (delta, theta, alpha, beta, gamma) during spontaneous state across groups (CON, n=5; MscL-G22S, n=5; MscL-G22N, n=5; MscS, n=5). **(E)** Comparison of detailed relative band-power distribution and theta/gamma band-power ratio among different groups (CON, n=5; MscL-G22S, n=5; MscL-G22N, n=5; MscS, n=5). Ordinary one-way ANOVA followed by Dunnett's multiple comparisons in **C**, Kruskal-Wallis one-way ANOVA on ranks followed by post-hoc Dunn's tests for multiple comparisons in **D** and **E**. The data are shown as mean ± SEM. * *P*<0.05, ** *P*<0.01, *** *P*<0.001, **** *P*<0.0001, ns, no significant. CON, empty virus injection group; MscL-G22S, represents the MscL-G22S channel group; MscL-G22N, represents the MscL-G22N channel group; MscS, represents the MscS channel group.

To examine how exogenously expressed mechanosensitive ion channels affect spontaneous neural activity, we recorded LFPs from isoflurane-anesthetized rat hippocampus following AAV-mediated channel expression, confirmed by confocal fluorescence imaging (Figure 2A).

Analysis of spontaneous neuronal activity revealed channel-specific baseline effects: MscS overexpression significantly increased averaged LFP waveform amplitude and total power versus control (MscS: 41.47±0.44 dB vs. CON: 36.66±0.54 dB, *P* < 0.0001), while MscL mutants reduced these parameters (MscL-G22S: 31.72±0.59 dB, *P* < 0.0001; MscL-G22N: 33.92±0.84 dB, *P*=0.0106; Figure 2B-C). These divergent effects indicate fundamentally different mechanisms of channel-membrane interaction even before ultrasound stimulation.

Our analysis of frequency bands was motivated by their distinct roles in hippocampal function and their sensitivity to neuromodulation-induced changes 51, 52. Frequency band analysis showed channel-specific oscillatory effects (Figure 2D), with MscL-G22N exhibiting increased delta power (60.29±4.09%, *P* < 0.0001), potentially indicating enhanced inhibitory states. Decreased higher frequency bands (theta: 20.57±0.80%, *P* = 0.0125; alpha: 8.51±1.44%, *P* < 0.0001; beta: 9.57±1.99%, *P* = 0.0004; gamma: 1.06±0.15%, *P* < 0.0001). Theta and gamma bands, critical for memory processing, were analyzed to assess the impact of channel expression on cognitive circuits, with the theta/gamma ratio serving as a marker of memory-related activity 53, 54 was significantly increased in the MscL-G22N group (25.24±2.87%, *P* = 0.0015, Figure 2E).

These findings demonstrate that mechanosensitive channels produce distinct electrophysiological signatures, suggesting channel properties, such as conductance, ion selectivity, and spontaneous opening probability, fundamentally alter network excitability, thereby providing a foundation for targeted neuromodulation approaches.

### Ultrasound-induced power changes in hippocampal neural activity mediated by different mechanosensitive channels

**Figure 3:**
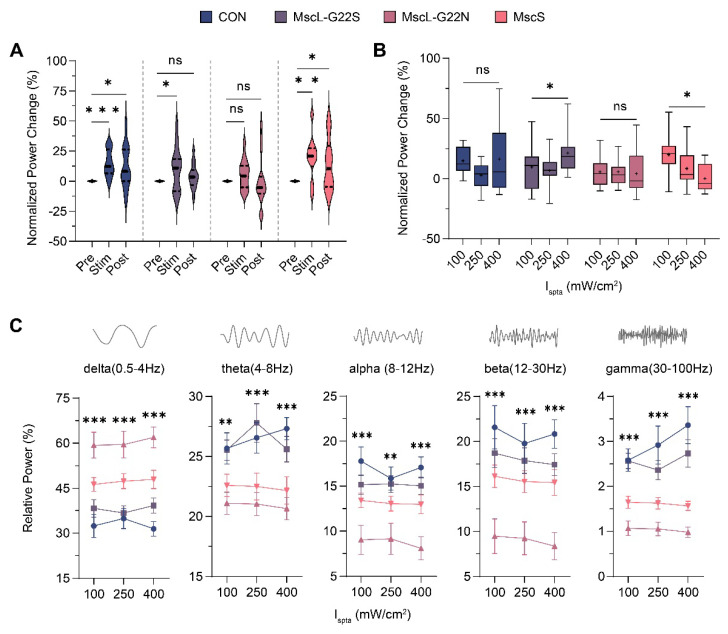
** Ultrasound**-**induced modulation of hippocampal LFP mediated by different mechanosensitive ion channels. (A)** Temporal evolution of normalized power changes in pre-stimulation, stimulation, and post-stimulation states across different mechanosensitive ion channel groups (CON, n=5; MscL-G22S, n=5; MscL-G22N, n=5; MscS, n=5). **(B)** Normalized power changes during the stimulation state across three ultrasound intensities (CON, n=5; MscL-G22S, n=5; MscL-G22N, n=5; MscS, n=5). **(C)** Relative power across frequency bands (delta, theta, alpha, beta, gamma) for different mechanosensitive ion channel groups at three ultrasound intensities, the statistical annotations information is displayed in Supplementary Table S5 (CON, n=5; MscL-G22S, n=5; MscL-G22N, n=5; MscS, n=5). Ordinary one-way ANOVA followed by Dunnett's multiple comparisons in **A**, Kruskal-Wallis one-way ANOVA on ranks followed by post-hoc Dunn's tests for multiple comparisons in **B** and **C**. The data are shown as mean ± SEM. * *P*<0.05, ** *P*<0.01, *** *P*<0.001, ns, no significant. CON, empty virus injection group; MscL-G22S, represents the MscL-G22S channel group; MscL-G22N, represents the MscL-G22N channel group; MscS, represents the MscS channel group.

After characterizing baseline activity, we investigated channel-specific responses to ultrasonic stimulation using normalized power changes across frequency bands and time-frequency analysis, with activity normalized to pre-stimulation baseline (Figure 3A). Our ultrasound stimulation protocol included pre-stimulation, stimulation, and post-stimulation; each phase lasted 150 s.

At 100 mW/cm² ultrasound stimulation, a significant increase in total power occurred in the CON, MscL-G22S, and MscS groups (CON: 14.92±3.07%, *P* = 0.0007; MscL-G22S: 9.71±4.50%, *P* = 0.047; MscS: 19.95±5.68%, *P =* 0.0065), whereas the MscL-G22N group showed minimal response (3.42±2.81%, *P* = 0.099). This finding is notable because MscL-G22N has the lowest reported mechanosensitivity threshold 35 in standard assays, yet showed the weakest ultrasound response, suggesting ultrasound sensitivity depends on factors beyond mechanical gating thresholds. After stimulation cessation, neural activity returned to baseline levels in all groups, demonstrating robust reversibility.

To systematically evaluate the ultrasonic neuromodulation mediated by exogenous channels, we varied ultrasound stimulation intensity (100, 250, and 400 mW/cm²) and observed channel-specific differences (Figure 3B): MscL-G22S exhibited a non-monotonic intensity-response relationship (100 mW/cm²: 9.71±4.50%; 250 mW/cm²: 6.67±2.96%; 400 mW/cm²: 21.2±4.29%; *P* = 0.0349), while MscS showed an inverse relationship (100 mW/cm²: 19.95±5.68%; 250 mW/cm²: 8.40±4.19%; 400 mW/cm²: 0.02±3.28%; *P* = 0.019). The CON and MscL-G22N groups showed no significant differences across intensities. These contrasting patterns suggest fundamentally different biophysical mechanisms governing channel responses to ultrasound.

Frequency-specific analysis revealed distinct ultrasound-induced modulation patterns across neural oscillation bands (Figure 3C, Supplementary Table S5). The CON group exhibited intensity-dependent increases in theta and gamma bands, whereas the MscL-G22N and MscS groups showed intensity-dependent decreases in these same bands. MscS particularly demonstrated a graded reduction in both theta and gamma power that correlated with increasing ultrasound intensity. While relative distribution among frequency bands remained consistent within each channel group across stimulation intensities, the relative power profiles differed significantly between channel types at the same intensity, revealing channel-specific spectral signatures.

These findings demonstrate that ultrasound stimulation modulates hippocampal neural activity in a channel-specific manner, with power changes correlating with both stimulation intensity and channel type. The distinctly different intensity-response relationships of MscL-G22S and MscS provide complementary tools for applications requiring either intensity-dependent enhancement or suppression of neural activity. The relative distribution across frequency bands is primarily determined by the specific channel variant, suggesting potential for frequency-selective neuromodulation through channel engineering.

### Ultrasound-evoked potentials reveal distinct response patterns across modified mechanosensitive channels

**Figure 4:**
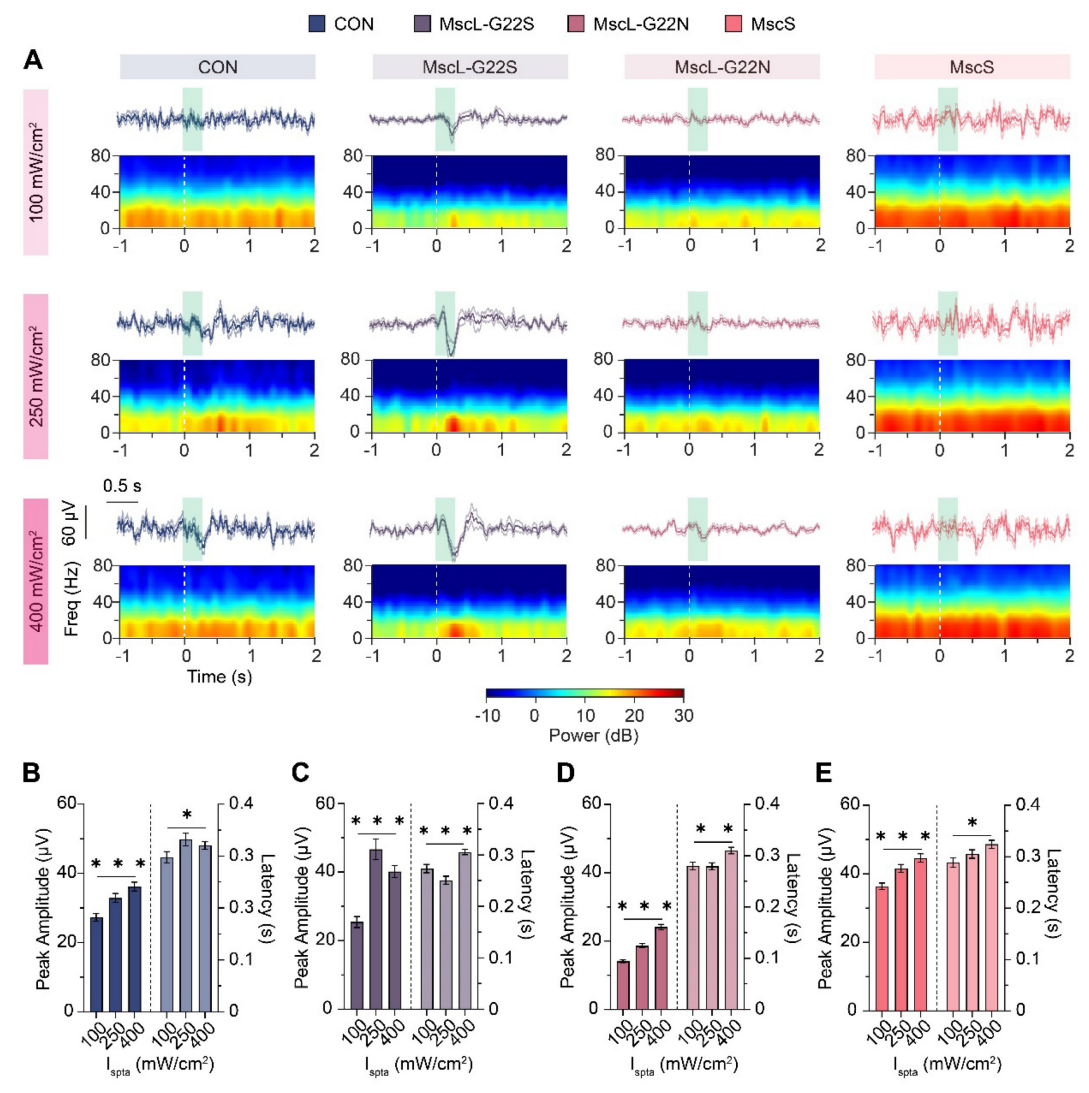
** Ultrasound-evoked potentials (UEPs) and quantified N1 responses for different mechanosensitive ion channels in the hippocampus. (A)** Average UEPs waveforms (top panel, bold line represents mean waveform across animals, light-colored lines above and below indicate the SEM across all animals within group, green square indicates ultrasound stimulation time) and corresponding time-frequency spectra (bottom panel, heatmap, white dotted line indicates ultrasound start time) across different ultrasound intensities for each mechanosensitive ion channel group (CON, n=5; MscL-G22S, n=5; MscL-G22N, n=5; MscS, n=5). The time window spans from -1 to 2 seconds relative to stimulus onset. **(B) ~ (E)** Quantification of the first negative peak (N1) amplitude and latency for different mechanosensitive ion channel groups across three ultrasound intensities.** (B)** CON (100 mW/cm², n=192; 250 mW/cm², n=128; 400 mW/cm², n=160), **(C)** MscL-G22S (100 mW/cm², n=184; 250 mW/cm²,n=169; 400 mW/cm², n=186), **(D)** MscL-G22N (100 mW/cm², n=214; 250 mW/cm², n=240; 400 mW/cm², n=206), and **(E)** MscS (100 mW/cm², n=186; 250 mW/cm², n=202; 400 mW/cm², n=204). Kruskal-Wallis test followed by Dunn's post-hoc test in **B** ~**E,** the data are shown as mean ± SEM. * *P*<0.05, ** *P*<0.01, *** *P*<0.001. CON, empty virus injection group; MscL-G22S, represents the MscL-G22S channel group; MscL-G22N, represents the MscL-G22N channel group; MscS, represents the MscS channel group.

To quantify immediate neuronal responses with millisecond temporal resolution, we measured UEPs, focusing on the first negative peak (N1) amplitude and latency across different channel types and stimulation intensities (Figure 4A-E).

UEPs were systematically evaluated across increasing ultrasound intensities (100, 250, and 400 mW/cm²) for all experimental groups, with average waveforms showing response consistency across trials (Figure 4A).

Channel-specific UEPs responses varied markedly across ultrasound intensities. At low intensity (100 mW/cm²), only the MscL-G22S group exhibited a detectable response, while CON, MscL-G22N, and MscS groups showed no significant evoked potentials. At moderate intensity (250 mW/cm²), the CON group displayed a modest response, while MscL-G22S showed a significantly stronger response, with MscL-G22N and MscS showing no significant waveforms. At high intensity (400 mW/cm²), both CON and MscL-G22S groups displayed prominent evoked waveforms (mean peak amplitudes: 36.2±1.3 μV and 40.16±1.7 μV, respectively), MscL-G22N exhibited a modest response (mean peak amplitude: 24.2±0.76 μV), while MscS still showed no significant evoked response. Notably, MscL-G22N, despite having the lowest mechanical activation threshold in previous channel gating research, did not demonstrate superior ultrasound sensitivity compared to G22S.

We quantified both latency (time to first negative peak) and peak amplitude within a 0-500 ms post-stimulation window. Quantitative analysis revealed distinct channel-specific patterns (Fig. 4B-E). In the control group, peak amplitude significantly increased with intensity, while latency showed significant shortening between lower and higher intensities. MscL-G22S demonstrated the most robust response profile, with peak amplitude increasing dramatically with intensity while latency decreased significantly, indicating enhanced ultrasound sensitivity. MscL-G22N showed a similar but more modest trend. MscS exhibited only modest increases in peak amplitude with intensity and minimal latency changes, indicating less efficient ultrasound coupling.

These results establish a clear hierarchy of channel-mediated ultrasound sensitivity (MscL-G22S > MscL-G22N > MscS) based on evoked potential characteristics. This electrophysiological dissociation between channels suggests different mechanisms for transient versus sustained ultrasound effects, with important implications for designing channels optimized for specific neuromodulation applications requiring either immediate or sustained neural modulation.

### Candidate mechanosensitive ion channels demonstrate favorable safety profile with preserved behavior and neural histology

The translational potential of engineered mechanosensitive channels depends critically on their safety profile. We comprehensively evaluated their impact on neural function, behavior, and tissue integrity using established behavioral paradigms and histological analyses.

**Figure 5:**
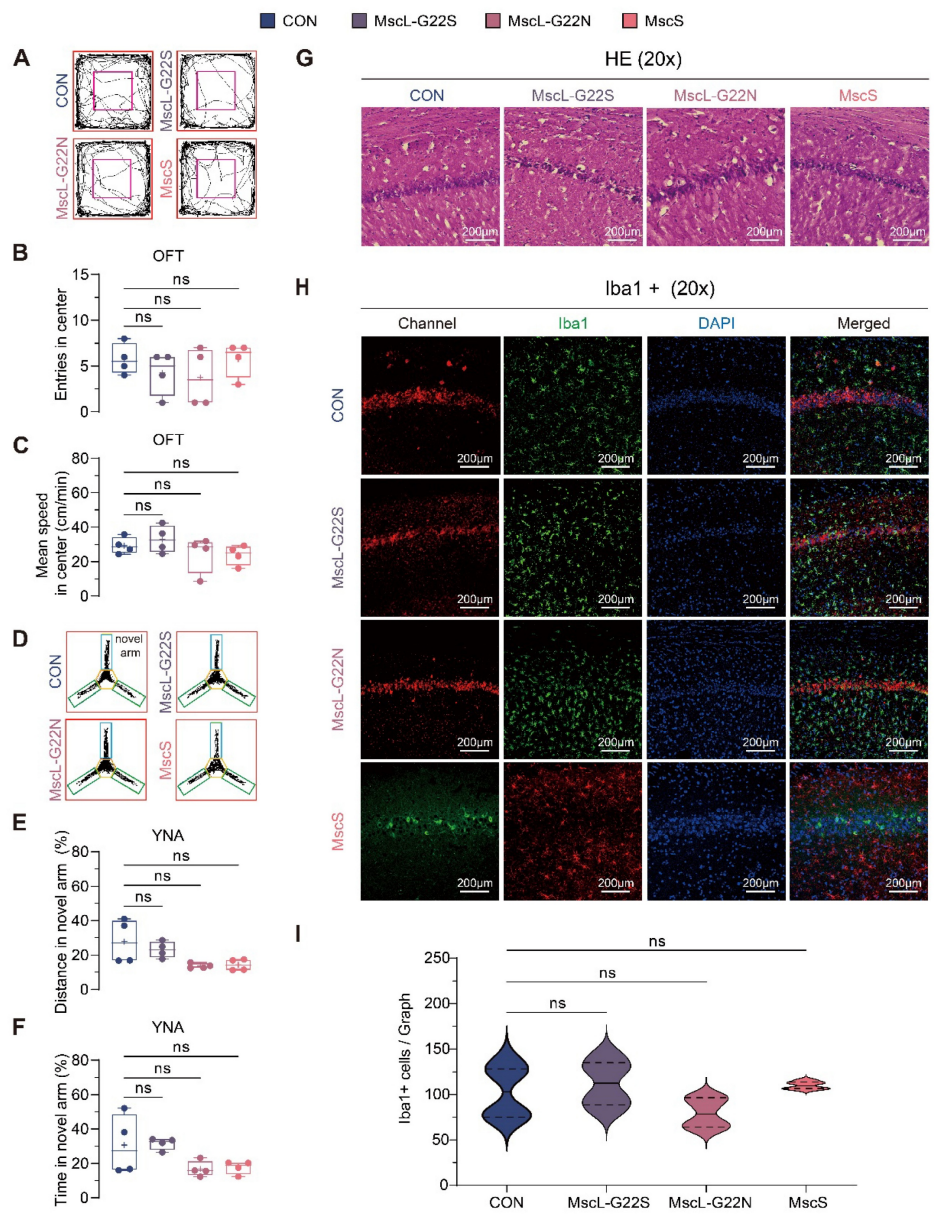
** Baseline behavior performance and histological assessment of mechanosensitive ion channels expression in the hippocampus. (A)** Representative movement trajectories in the open field test (OFT). **(B, C)** The number of center entries and mean speed in the center during OFT (CON, n=4; MscL-G22S, n=4; MscL-G22N, n=4; MscS, n=4). **(D)** Representative movement trajectories in the Y-maze novel arm (YNA) test. **(E, F)** Percentage of distance traveled and time spent in the novel arm during the YNA test (CON, n=4; MscL-G22S, n=4; MscL-G22N, n=4; MscS, n=4). **(G)** Representative images of the hippocampal CA1 region stained with hematoxylin-eosin (Images at 20x magnification, scale bar: 200 μm).** (H)** Representative confocal fluorescence images of the hippocampal CA1 region showing microglial marker Iba1-positive cells (green in CON, MscL-G22S, MscL-G22N; red in MscS, DAPI-stained nuclei (blue), images at 20x magnification, scale bar: 200 μm). **(I)** Quantification of Iba1-positive microglia cells were measured using ImageJ (CON, n=6; MscL-G22S, n=6; MscL-G22N, n=6; MscS, n=6). Kruskal-Wallis test followed by Dunn's post-hoc test in **B, C, E, F, and I.** The data are shown as mean ± SEM. ns, no significant. CON, empty virus injection group; MscL-G22S, represents the MscL-G22S channel group; MscL-G22N, represents the MscL-G22N channel group; MscS, represents the MscS channel group.

After 4 weeks of expression, all experimental groups showed anxiety-related behaviors comparable to controls in both open field test (OFT) and elevated plus maze (EPM), with no significant differences in center entries (Figure 5B), center speed (Figure 5C), distance measures (Supplementary Figure S7A-B), or EPM parameters like distance in open arm (Supplementary Figure S8B-D), confirming that channel expression does not significantly alter anxiety-related behaviors.

Assessment of depressive-like behaviors using the forced swim test (FST) revealed that MscL-G22S and MscL-G22N groups showed no significant differences compared to controls. However, the MscS group displayed increased immobility number (*P* = 0.0169; Supplementary Figure S9A) and duration (*P* = 0.001; Supplementary Figure S9B), suggesting selective effects on stress responses potentially mediated through altered hippocampal excitability.

Next, Y-maze testing revealed no significant impairment in spatial recognition or working memory, with all channel groups showing performance comparable to controls in novel arm recognition (Figure 5E-F, Supplementary Figure S10A-B) and spontaneous alternation (Supplementary Figure S10C), indicating preserved cognitive function despite altered hippocampal electrophysiology.

Our baseline behavioral assessments demonstrated no significant large-scale phenotypic changes were observed at the current sample size, confirm that expression of mechanosensitive ion channels does not impair short-term spatial learning and memory, consistent with prior neuromodulation studies^24^, ^55^ While channel expression may induce subtle changes in LFP patterns (Figure 2B), these alterations likely reflect localized network adaptations that do not disrupt the broader hippocampal-cortical circuits required for behavioral tasks^56^, ^57^.

Beyond behavioral analyses, we also conducted histological examinations to evaluate the impact of channel overexpression on neuronal morphology and inflammatory responses. H&E staining of hippocampal CA1 (Figure 5G) showed no morphological alterations in neuronal architecture, with maintained pyramidal layer cell arrangement and normal nuclear morphology across all groups. Iba1 immunofluorescent staining revealed no significant differences in microglial cell numbers between channel-expressing and control groups (Figure 5H-I), indicating an absence of neuroinflammatory responses.

Collectively, these behavioral and histological analyses demonstrate that engineered mechanosensitive ion channels exhibit favorable biocompatibility with neural tissue, showing minimal evidence of toxicity, inflammation, or functional impairment at 4 weeks post-expression. This safety profile supports their potential for translation to neuromodulation applications.

### Different mechanosensitive ion channels drive channel-specific transcriptomic changes in hippocampal neurons

**Figure 6:**
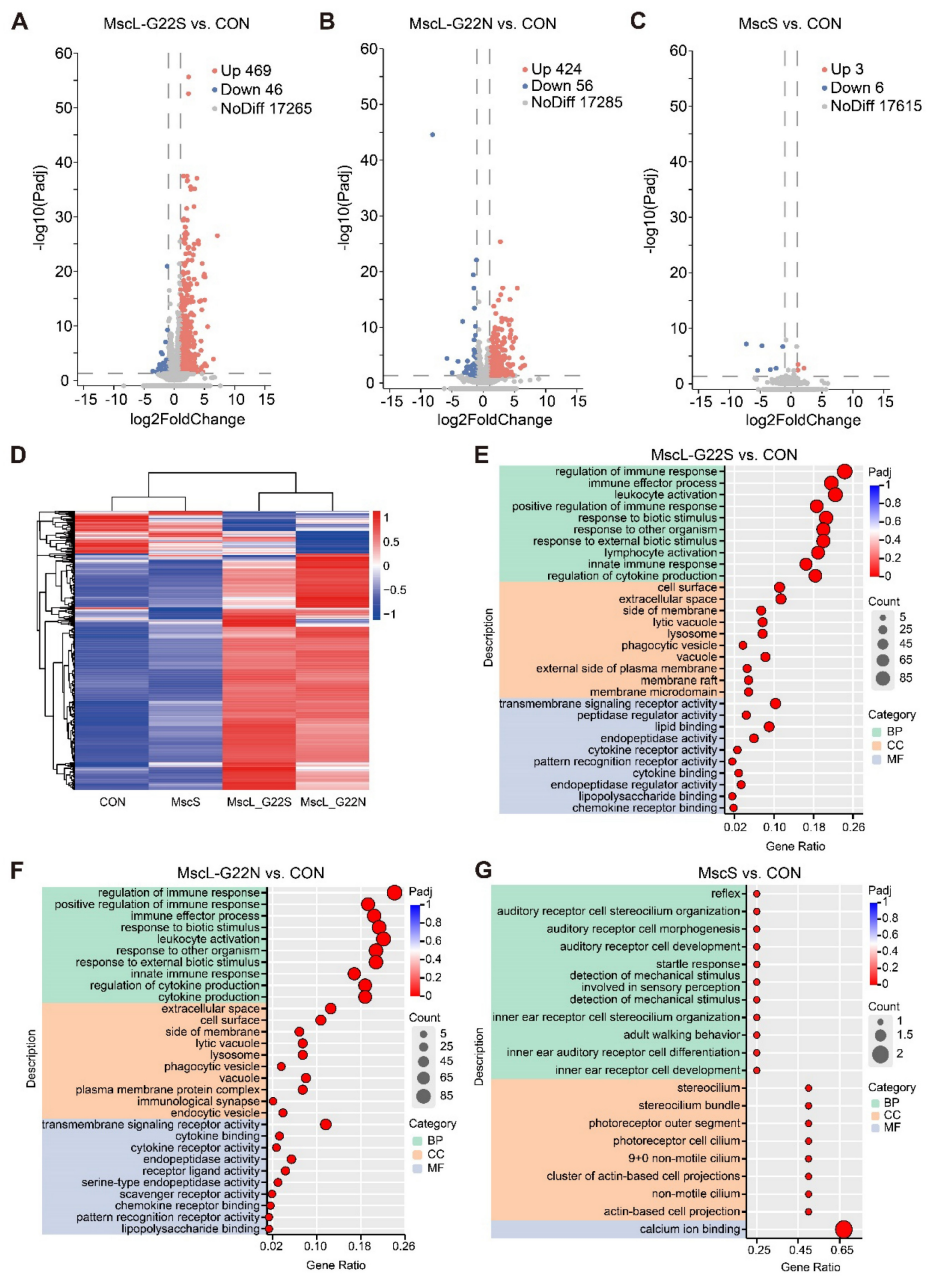
** Transcriptomic analysis of hippocampal tissue expressed with different mechanosensitive ion channels. (A-C)** Volcano plots showing differentially expressed genes (DEGs), defined by* Padj*≤ 0.05, |log2FC|≥1. in MscL-G22S, MscL-G22N, and MscS (CON, n=3; MscL-G22S, n=3; MscL-G22N, n=3; MscS, n=3). **(D)** Hierarchical clustering heatmap showing expression patterns of DEGs across all groups. **(E-G)** Gene Ontology (GO) enrichment analysis shows the top ten enriched terms for DEGs in (E) MscL-G22S, (F) MscL-G22N, and (G) MscS groups. Up, upregulated; Down, downregulated; NoDiff, no significant difference; BP, biological process (green shadow); CC, cellular component (orange shadow); MF, molecular function (blue shadow). CON, empty virus injection group; MscL-G22S, represents the MscL-G22S channel group; MscL-G22N, represents the MscL-G22N channel group; MscS, represents the MscS channel group.

To systematically evaluate the baseline transcriptional effects of chronic, ectopic channel expression alone on hippocampal tissue, and how channel overexpression affects endogenous gene expression, we conducted transcriptomic profiling via RNA-Seq. Differential gene expression analysis over 17,000 expressed genes revealed striking differences between channel types (Figure 6, A-C). MscL variants induced substantial transcriptional changes, with MscL-G22S showing 469 significantly upregulated and 46 downregulated genes, while MscL-G22N exhibited 424 upregulated and 56 downregulated genes (*Padj*≤ 0.05, |log2FC|≥1). In contrast, MscS overexpression produced remarkably minimal transcriptional alterations, with only 3 upregulated and 6 downregulated genes compared to controls (*Padj* ≤0.05, |log2FC|≥1). This dramatic difference highlights fundamental distinctions in how these channel families interact with cellular transcriptional machinery despite their shared mechanosensitivity.

Hierarchical clustering (Figure 6D) confirmed that MscS expression profiles closely resemble controls. Gene Ontology analysis (Figure 6E-G) revealed that MscL variants primarily affected immune responses, biotic stimuli, cell surface components, and transmembrane signaling, while MscS-altered genes were enriched for mechanoelectrical transduction and calcium ion binding in sensory cells.

These results demonstrate fundamentally different impacts: MscL variants induced broad changes affecting multiple cellular processes, while MscS exerted a highly targeted influence. Notably, MscS upregulated Calm2, a critical calcium-binding protein that modulates neuronal excitability through CaMKII, calcineurin, and various ion channels—potentially explaining observed electrophysiological and behavioral effects despite minimal transcriptional disruption. MscL-G22S and MscL-G22N displayed different electrophysiological responses and ultrasound sensitivity, but a highly similar number and type of differentially expressed genes. We hypothesize that this apparent difference between the functional and molecular phenotypes stems from a fundamental distinction in the timescale and nature of the measured phenomena.

## Discussion

This study advances our understanding of the biophysical mechanisms underlying ultrasound-mediated neuromodulation and provides insights toward more precise neuromodulation tools for potential therapeutic applications. By systematically characterizing the electrophysiological effects of exogenously expressed mechanosensitive channels in the hippocampus, we have identified distinct channel-specific response signatures that could facilitate the development of targeted approaches to non-invasive neuromodulation. Current therapeutic approaches for neurological disorders face limitations in pharmacological delivery, off-target effects, and surgical invasiveness. While conventional neuromodulation techniques like TMS and tDCS have clinical utility, they lack spatial precision and depth penetration for targeting deep brain structures 58. Ultrasound stimulation, with its superior depth penetration 59 and spatial resolution (1-5 mm compared to centimeters for TMS), represents a promising direction for non-invasive neuromodulation development. Our comparative analysis of multiple engineered channels revealed that classical mechanical sensitivity properties do not directly correlate with ultrasound responsiveness, a finding with implications for the design of sonogenetic actuators and understanding ultrasound-tissue interactions.

Sonogenetics—combining ultrasound with genetically encoded ultrasound-sensitive targets—addresses precision limitations of conventional ultrasound through enhanced cellular specificity 13, 24, 60-62. This approach enables more precise control of neural activity with potential therapeutic applications across multiple neurological disorders. However, the field has been constrained by limited available ultrasound-sensitive ion channels and an incomplete understanding of their comparative properties, including their distinct neuromodulatory signatures, intensity-response relationships, and biosafety profiles. Our research provides comparative data on engineered bacterial mechanosensitive channels' ability to mediate ultrasound neuromodulation with distinct characteristics—information that contributes to the development of targeted neuromodulation approaches.

Our findings demonstrate that different mechanosensitive channels provide distinct neuromodulatory signatures. At baseline, MscS increased the total power (Figure 2C) of hippocampal activity while MscL mutants reduced it—revealing differential impacts on intrinsic neural excitability patterns. These channels exhibited characteristically different responses to ultrasound across intensity parameters, with ultrasound-evoked potentials showing channel-specific signatures in amplitude, latency, and frequency distribution. These distinct profiles suggest a potential for selective modulation of neural oscillatory patterns relevant to cognitive processing and pathological states.

Importantly, all tested channels demonstrated favorable safety profiles with minimal effects on animal behavior, neuronal morphology, and inflammatory responses. The absence of significant microglial activation and preservation of cognitive function indicate these engineered channels may be integrated into neural circuits with limited adverse effects, an important consideration for future research applications.

### Channel-specific modulation of hippocampal neural activity

An important contribution of this research is identifying channel-specific differences in hippocampal neural activity modulation that could be exploited for targeted neuromodulation approaches. The differential effects on baseline neural activity—MscL mutants decreasing baseline power while MscS increasing it—highlight their nuanced roles in neural regulation, likely stemming from their distinct biophysical properties, including conductance, ion selectivity, and gating kinetics 63.

Our inclusion of the G22N variant, which possesses the lowest mechanical threshold among characterized G22 mutants, revealed an important biophysical insight: ultrasound sensitivity does not correlate linearly with established mechanical activation thresholds. Despite G22N's superior mechanical sensitivity in membrane tension assays, it showed inferior ultrasound responses compared to G22S. This finding suggests that ultrasound-channel interactions involve complex biophysical parameters beyond simple mechanical gating thresholds, potentially including channel kinetics, membrane coupling efficiency, and lipid environment interactions. This functional divergence was further evidenced by distinct changes in power distribution across frequency bands, particularly the significant increase in delta band power in the MscL-G22N group, suggesting altered synchronization properties in neuronal populations.

Ultrasound-mediated neuromodulation revealed differences in intensity-response relationships between channels: MscL-G22S exhibited enhanced responses with increasing ultrasound intensity 30, 32, while MscS showed an inverse relationship—suggesting potential for engineering stimulus-specific response profiles. This biophysical diversity may allow for selective targeting of circuit components with calibrated stimulation parameters, potentially improving control over neural network activity.

The observed differential modulation across frequency bands has implications for cognitive research, as these oscillations underpin aspects of memory formation and information processing in the hippocampus 64. The alterations in theta/gamma ratio are noteworthy, given their role in hippocampal information coding. The observed frequency-specific modulation capability suggests potential for investigating disorders characterized by aberrant neural synchrony, such as epilepsy and Alzheimer's disease, which are difficult to address with conventional pharmacological approaches.

Ultrasound-evoked potential analysis showed that mechanosensitive channel-mediated responses offer good temporal resolution with rapid response characteristics—a critically useful feature for applications requiring timing control, such as the study of epileptiform activity 65-67 or oscillations in movement disorders 68-71.

The key response parameters (amplitude and latency) showed dependence on ultrasound stimulation parameters with channel-specific sensitivity profiles: MscL-G22S-expressing neurons exhibited detectable evoked potentials at moderate intensities (250 mW/cm², Figure 4A second column), whereas MscS showed noticeable responses only at relatively higher intensities (400 mW/cm²; Figure 4A last column). This differential sensitivity suggests possibilities for selective circuit activation using intensity-based targeting—an advantage over conventional approaches that typically lack such parametric specificity. These distinct electrophysiological signatures provide a foundation for the future development of application-specific channel variants with tailored response characteristics. Although these significant results indicated that different mechanosensitive channels have unique mediation effects, limitations remain exist that given the difference in promoters and fluorescent reporters, potential variation in neuronal subtype targeting or reporter brightness cannot be excluded and the precise distribution responses across neuronal subtypes was not determined, future work will apply matched promoters and Cre-based strategies to isolate promoter- and cell-type-specific contributions.

### Favorable safety profile of mechanosensitive channel expression

An important finding for potential clinical translation is the favorable safety profile of these mechanosensitive channels when expressed in the hippocampus, extending previous observations of bacterial channels in mammalian systems 72, 73. Our comprehensive behavioral and histological analyses showed minimal effects on anxiety, spatial cognition, neuronal morphology, and inflammatory responses—addressing essential safety considerations for genetic neuromodulation approaches. While MscS expression produced mild effects on depression-like behaviors in the forced swim test (potentially reflecting its modulation of hippocampal theta oscillations implicated in stress responses), this occurred without corresponding histological changes or deficits in other domains, suggesting specific functional modulation rather than neurotoxicity. Although MscS may not be efficiently gated by ultrasound, its constitutive or spontaneous activity within the neuronal membrane could subtly alter the basal membrane potential and excitability of hippocampal neurons. This chronic, low-level perturbation could influence the overall state of the hippocampal circuit, which is a known substrate for depression. Given the current sample size (n = 4 per group) and a two-sided α = 0.05 design, the study was powered to detect large effect sizes but may have limited sensitivity to subtle or moderate differences. The behavior measurements displayed there non-significant different should be interpreted as no large effects were observed.

While our histological analyses confirmed the absence of overt cell death or neuroinflammation, they did not assess potential finer-scale structural alterations. Key metrics such as dendritic arborization, spine density, and synaptic protein expression were not measured, and we recognize that such subtle synaptic remodeling could occur with chronic channel expression. Future investigations incorporating these detailed structural analyses are essential to fully characterize the chronic impact of this neuromodulation strategy on synaptic and circuit integrity, providing a more complete picture of its safety profile for long-term applications.

This favorable safety profile is noteworthy given the hippocampus's vulnerability to excitotoxicity and its role in cognition. The preservation of neural function and tissue integrity over the four-week expression period suggests these engineered channels can be safely integrated into neural circuits for research on ultrasound-mediated neuromodulation. The absence of significant adverse effects addresses a critical requirement for clinical applications and supports further development of this technology.

### Impact of anesthesia on electrophysiological results

A methodological limitation of our study is the use of light isoflurane anesthesia during electrophysiological recordings, which suppresses high-frequency oscillations. Thus, frequency-specific modulation patterns are interpreted within the anesthetized state rather than the awake state. Isoflurane alters LFP frequency distributions by enhancing lower frequencies while suppressing higher frequencies 74-77, potentially influencing both baseline activity and ultrasound-induced changes. However, existing research indicates that isoflurane does not fundamentally confound the neuromodulatory effects of ultrasound stimulation 78, 79. Multiple studies have demonstrated that ultrasound can effectively modulate neural activity even under anesthesia 80, with effects occurring independently of anesthesia-induced baseline shifts, suggesting direct ultrasound-neural tissue interactions 81-83. To fully validate these findings for clinical applications, future studies should employ alternative anesthesia protocols or conduct experiments in awake animals to assess efficacy during active cognitive processing.

### Transcriptomic changes and biosafety considerations

Our transcriptomic analysis revealed an important finding: MscL variants induced substantially broader transcriptional changes (affecting hundreds of genes) compared to MscS (altering only 9 genes). This difference in genomic impact has implications for selecting appropriate channels for specific applications.

The extensive transcriptional changes associated with MscL variants likely stem from their large pore diameter (~2 nm when fully open), which permits the passage of not only ions but also small biological molecules including ATP 84, 85. This non-selective permeability could trigger alterations in cellular metabolism and activate stress response pathways even at sub-threshold states. Gene ontology analysis confirmed that MscL variants primarily affected immune-related processes and signaling pathways, consistent with cellular responses to potential metabolite leakage. Additionally, heterologously expressed MscL channels may function differently in eukaryotic membranes compared to their native bacterial environment, potentially exhibiting altered tension sensitivity thresholds or incomplete closure 86.

MscS's remarkably smaller impact on gene expression likely reflects its smaller conductance (~1 nS versus ~3 nS for MscL), higher ion selectivity, and tighter regulation of gating 87. This substantial difference in transcriptional impact suggests MscS may be advantageous for applications requiring minimal disruption of baseline cellular functions, particularly for longer-term studies. The primary MscS-induced change—upregulation of Calm2, encoding calmodulin-2 88, 89, involved in various intracellular signaling cascades, including those related to stress and neuronal excitability, may explain the observed alterations in spontaneous activity and depressive-like behavior, warrants further investigation, but represents a significantly more targeted effect than the broad changes induced by MscL variants. Future research should focus on engineering MscS variants with enhanced ultrasound sensitivity while maintaining their favorable biosafety profile, potentially offering an improved balance of efficacy and safety for neuromodulation studies.

### Long-term monitoring and therapeutic potential

While our study provides insights into effects over four weeks, longer-term monitoring would be valuable for understanding potential chronic applications. Understanding the long-term consequences of channel expression and repeated ultrasound activation regarding neuroplasticity, homeostatic compensation, and adaptation would be important for evaluating potential applications in chronic conditions. For research applications with limited timeframes, our findings support biosafety and functional stability. Further research would need to address expression stability, potential immunogenicity of bacterial proteins, and safety assessments over longer periods. We concur those subsequent longitudinal studies in awake, behaving subjects and validation in appropriate disease models will be indispensable for substantiating the translational potential of these mechanosensitive channels for clinical applications in non-invasive neuromodulation.

Future work should consider strategies to enhance long-term stability and reduce potential immunogenicity, such as codon optimization, incorporation of mammalian trafficking signals, or development of chimeric channels combining bacterial mechanosensitive domains with mammalian channel backbones.

### Future implications and breakthroughs

This study provides biophysical insights into ultrasound-mediated neuromodulation and suggests avenues for both basic neuroscience research and potential applications. Our findings suggest that engineered mechanosensitive channels may selectively modulate specific frequency bands of neural activity, offering new approaches for investigating oscillatory patterns relevant to neurological and psychiatric disorders. The channel-specific intensity-response relationships we identified suggest possibilities for calibrating neuromodulatory effects, potentially offering advantages over current approaches.

The integration of engineered mechanosensitive channels with non-invasive ultrasound stimulation combines the spatial precision of ultrasound with the cell-type specificity of genetic targeting—a promising approach for investigating neural circuits. Our comparative analysis of channel variants revealed that standard measures of mechanosensitivity do not directly predict ultrasound responsiveness, indicating that the development of sonogenetic actuators should incorporate both mechanical threshold measurements and ultrasound-specific response characteristics.

Beyond therapeutic applications, this technology offers capabilities for probing neural connectivity patterns in intact circuits with improved depth penetration compared to some existing techniques. Potential future applications might include the investigation of conditions with localized activity, such as focal epilepsy, hippocampal sclerosis, or circuit-specific disorders, where modulation of defined neural circuits could provide insights while minimizing off-target effects.

In summary, this study provides a direct electrophysiological comparison of three distinct mechanosensitive channel constructs for sonogenetics. By defining the time-locked UEPs amplitude as the primary outcome, we definitively characterized their differential activation efficacy *in vivo*. Our secondary analyses of LFP oscillations further revealed corresponding differences in their downstream modulation of hippocampal network activity. Finally, while the exploratory behavioral and transcriptomic data were limited by sample size and construct differences, they provide a valuable preliminary foundation for future work.

This hierarchical approach clarifies the specific contributions of each construct and provides a necessary framework for the rational design of future precision sonogenetic tools. Ultimately, this research contributes to developing non-invasive neuromodulation strategies with improved precision and specificity, potentially enhancing neuroscience research capabilities and informing future approaches for neurological and psychiatric disorders.

## Conclusion

Our comparative analysis of mechanosensitive channels reveals distinct electrophysiological signatures that provide targeted approaches for ultrasound neuromodulation. Different channels exhibited distinct modulation properties, with MscL variants and MscS providing complementary neuromodulation capabilities for various applications. This channel-specific toolkit enables customized sonogenetic approaches for targeting circuit-specific oscillatory dysfunction in neurological disorders. These findings provide a roadmap for developing precision sonogenetics applications with appropriate channel selection based on specific therapeutic needs.

## Supplementary Material

Supplementary methods, figures, and tables.

## Abbreviations

MscL: large conductance mechanosensitive ion channel
MscS: small conductance mechanosensitive ion channel
AAV: adeno-associated virus
LFP: local field potential
UEPs: ultrasound evoked potentials
FF: fundamental frequency
PRF: pulse repetition frequency
DC: duty cycle
TBD: tone burst duration
SD: stimulation duration
ISI: inter-stimulation intervals
Iba1: ionized calcium-binding adapter molecule 1
OFT: open field test
EPM: elevated plus maze
YNA: Y-maze novel arm
YSA: Y-maze spontaneous alternation
FST: forced swim test
