# Supplementary material for: Channel-specific differential effects of bacterial mechanosensitive channels for ultrasound neuromodulation in precision sonogenetics
Source: Theranostics. 2026 Jan 1;16(5):2447–65. doi: 10.7150/thno.119770 (PMC12712913; doi:10.7150/thno.119770)
Supplement: Supplementary file 1 — Supplementary methods, figures, and tables. [file thnov16p2447s1.pdf]

Supplementary material

Materials and methods

Virus packaging

Supplementary Table S1. The MS channel AAV virus construction information.

MS channel	Virus sample	Serotype	Titer （v.g./ml）
CON	pAAV-hSyn- linker-mCherry-3xFLAG-WPRE	AAV9	2.23E+13
MscL-G22S	pAAV-hSyn-MscL_G22S-linker-mCherry-3xFLAG-WPRE	AAV9	8.36E+12
MscL-G22N	pAAV-hSyn-MscL_G22N-linker-mCherry-3xFLAG-WPRE	AAV9	1.12E+13
MscS	pAAV-CaMKIIa-MscS_ECOLI-P2A-GCaMP6m-WPRE	AAV9	1.39E+13

Experimental design and blinding procedures

To control confounding variables and to avoid subjective bias, randomization and blinding procedures were used across all experiment designs and the data analysis stage. Randomization: Animals were randomly allocated to the four experimental groups before any viral injections. For baseline behavioral testing, this randomization ensured comparability between groups from the outset. For electrophysiological recordings, the order of experiments for animals within the same group was also randomized to control for potential time-dependent variables.

Blinding Procedures: We implemented blinding at the critical stages of data analysis and quantification to prevent observer bias. The specific steps were as follows: (1) Virus Injection and Tissue Processing: The experimenters performing the virus injections, as well as the subsequent histological slicing and staining, were aware of the group assignments to ensure correct sample handling. (2) Behavioral Data Analysis: Blinding was strictly enforced during the analysis of behavioral data. The raw data were coded and re-numbered before being handed to the analyst. The analyst

remained fully blinded to the group assignments until all statistical analyses were complete. (3) Electrophysiological Data Recording and Analysis: g: Recordings were performed in random order within each group. The analysis of electrophysiological data followed a highly standardized and objective pipeline. All recordings were subjected to identical filtering and 50 Hz noise removal. Signals from 16 channels were averaged within animals and subsequently averaged across animals within each group for presentation. (4) Histology Quantification: While the experimenter processing the tissues knew the groups, the quantification step was performed under blinded conditions. The fluorescence quantification and cell counting were conducted independently in a blinded fashion, using coded images and consistent thresholding criteria across all samples.

Ultrasound calibration and estimation parameters

For in-vivo ultrasound neuromodulation, safety profile is standard and essential for reproducibility. Based on the ultrasound stimulation parameters which used in this study, fundamental frequency (FF) of 1.0 MHz, a pulse repetition frequency (PRF) of 1.0 kHz, a duty cycle (DC) of 50%, a 0.5 ms tone burst duration (TBD), a 0.3 s stimulation duration (SD), and inter-stimulation intervals (ISI) of 3 s. All the other required calibration and estimation parameters which involved in the experiment were reported in the following table.

Supplementary Table S2. Ultrasound calibration and estimation parameters

**PNP（MPa）**	**MI**	**I_{sppa} (mW/cm^2)**	**I_{spta} (mW/cm^2)**	**ΔT_{max} (°C, 50 pulses)**
0.15	0.15	200	100	0.046
0.25	0.25	500	250	0.114
0.35	0.35	800	400	0.182

Our maximum ultrasound parameters (PNP = 0.35 MPa, Ispta = 400 mW/cm^2, Isppa = 800 mW/cm^2; MI = 0.35 < FDA limit 1.9) indicate negligible cavitation risk. The estimated temperature rise after 50 pulses (150 s) was < 0.2 °C, confirming thermal and mechanical safety under all tested conditions.

Results

Artifact-exclusion controls

A post-mortem control experiment and sham stimulation control experiment were conducted to explicit ultrasound artifact.

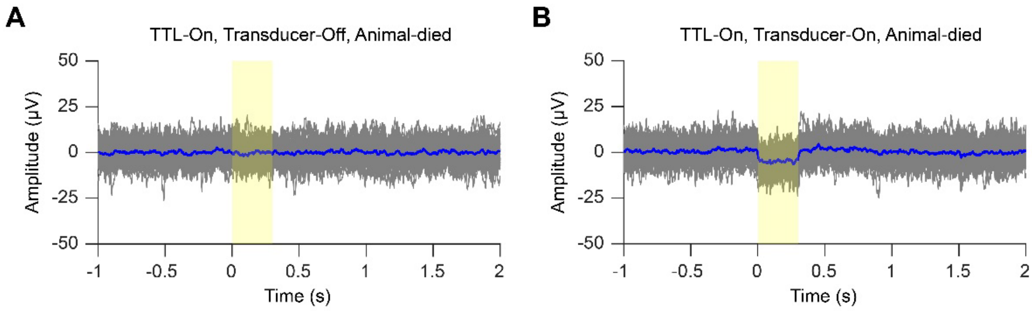

Supplementary Figure S1. **Post-mortem control in a euthanized animal. (A)** Mean waveform under TTL-on condition. **(B)** Mean waveform with ultrasound transducer-on condition. The gray part represents each TTL sync waveform trace, the blue wave is the average trace of 50 pulses, the yellow shadow represents the ultrasound stimulation time of 0.3 s.

After recording the baseline waveform, the animal was euthanized. We immediately continued to deliver the identical ultrasound stimulation protocol to the animal post-mortem. When ultrasound was applied, there was a small negative square wave was recorded in sync with the timestamp (Supplementary Figure S1B). This character wave was used to recognize the artifact in the data analysis stage. This experiment allowed us to record a high-fidelity template of the non-biological, low-frequency artifact generated by the ultrasound system in the absence of any neural activity.

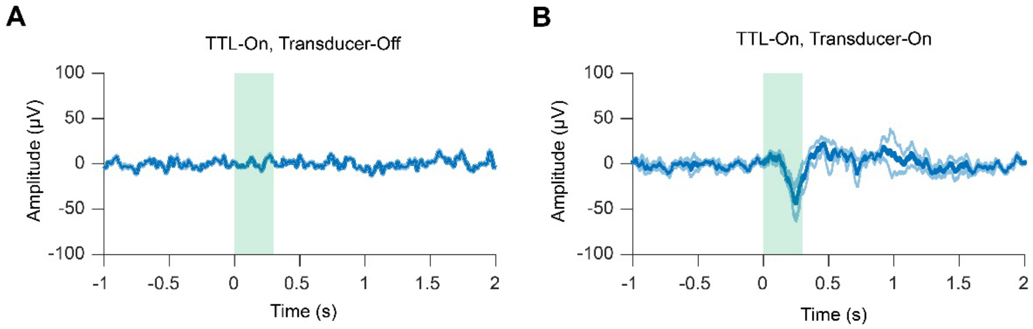

Supplementary Figure S2. Sham control in a living animal. (A) Average waveforms with TTL-on & Transducer-off. **(B)** Average UEPs waveforms with TTL-on & Transducer-on. The

bold line represents the mean waveform across channels, light-colored lines above and below indicate the SEM, green square indicates ultrasound stimulation time.

To further confirm the UEPs, in living anesthetized animals, we compared the signal between two conditions: TTL-on & Transducer-off (sham stimulation, Supplementary Figure S2) versus TTL-on & Transducer-on (real stimulation, Supplementary Figure S2B). We confirmed that the UEP response was absent in the 'Transducer-off' condition, definitively proving that the observed potential was contingent on the acoustic energy delivery and not an electrical artifact from the TTL trigger signal.

Ultrasound thermal effect

To eliminate the thermal effect confound, we performed a simulation-based thermal analysis using parameters that match those applied in our in vivo experiments, calibrated to the acoustic properties of brain tissue rather than water. The temperature elevation was estimated according to standard bio-heat transfer equations under continuous exposure conditions, representing the upper-bound scenario. The results were summarized in the table below.

Supplementary Table S3. Estimation results of maximum temperature change.

**PNP（MPa）**	**I_{spta} (mW/cm^2)**	**ΔT_{max} (°C) / pulse**	**ΔT_{max} (°C, 50 pulses)**
0.15	100	0.00096	0.046
0.25	250	0.00240	0.114
0.35	400	0.00384	0.182

The calculated maximum temperature rise per 0.3 s burst was <0.004 °C, and even after 50 pulses (150 s total exposure), the cumulative temperature increases at the acoustic focus (Z = 0 mm) remained below 0.2 °C across all intensities tested.

To further illustrate the spatial distribution of heating, supplementary figure S3 shows the simulated temperature rise along the ultrasound propagation axis (Z-direction). The results indicate that ΔT rapidly decreases with increasing depth from the focal region, with less than 10% of the peak value beyond 10–15 mm.

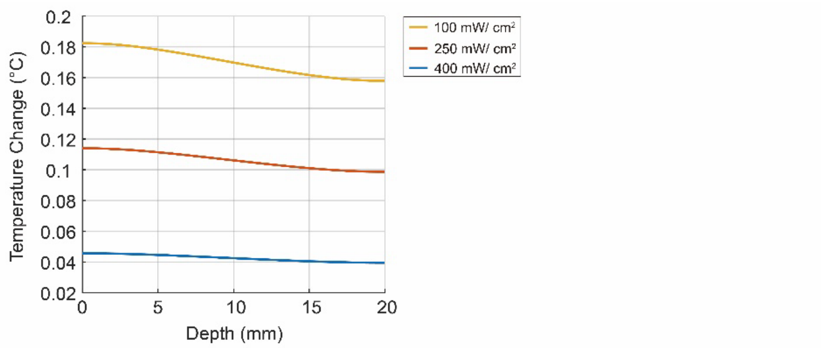

Supplementary Figure S3. Simulated temperature rise along the acoustic propagation axis in brain tissue under different acoustic intensities (yellow:100 mW/cm², red: 250 mW/cm², blue:400 mW/cm²).

The maximum temperature change (<0.2 °C) occurs at the focal region and decays rapidly with depth, indicating negligible thermal accumulation during the stimulation protocol.

In addition, we are conducting temperature measurements (Supplementary Figure S4) in a degassed water tank using a fine thermocouple placed at and around the acoustic focus under identical ultrasound parameters. The experimental temperature profiles displayed below (Supplementary Table 4) were used to experimentally verify the negligible heating effect predicted by the theoretical model.

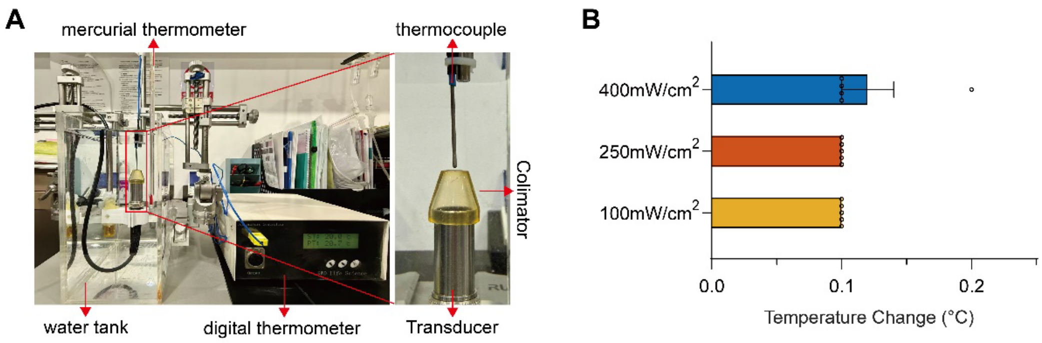

Supplementary Figure S4. Temperature measurements diagram and result. (A) Temperature measurement setting in the degassed water tank. **(B)** Temperature measurement results under different ultrasound stimulation intensities.

Supplementary Table S4. Measurement of temperature change in the water tank.

PNP（MPa）	I_{spta} (mW/cm^2)	ΔT_{max} (°C, 50 pulses)
0.15	100	0.1
0.25	250	0.1
0.35	400	0.2

Non-monotonic dose–response relationship with subject-level data

To reduce the influence of group averaging, we re-examined the raw, per-animal dose-response trajectories for all subjects in all groups. The per-animal spaghetti plots below (Supplementary Figure S5), showing that the reduced response at 250 mW/cm^2 for MscL-G22S is reproducibly observed across animals rather than arising from group-averaging artifacts.

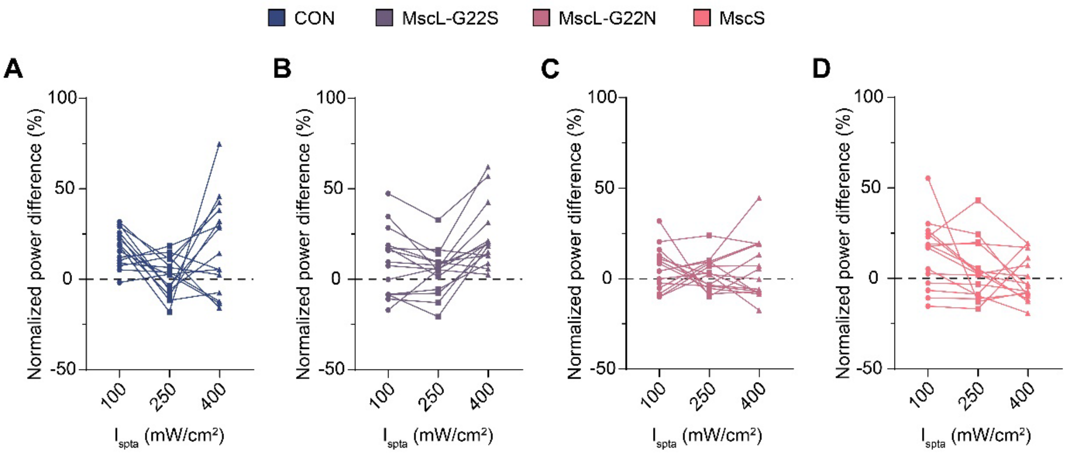

Supplementary Figure S5. (A)~(D) The normalized power difference spaghetti plots (per-animal across 3 trails) of different groups.

Relative band-power change statistical summary

Supplementary Table S5. Statistical results of relative band-power changes during ultrasound stimulation.

Band	Group	Intensity	mean	SEM	p-value
Delta	CON	100 mW/cm^2	32.4%	3.9%	*P*=0.0001,***
	MscL-G22S		38.2%	2.9%	ε^2=0.293
	MscL-G22N		59.3%	4.3%	95% CIs = [-45.49, -10.73]
	MscS		46.2%	2.3%	
	CON	250 mW/cm^2	34.9%	3.4%	*P*=0.0001,***
	MscL-G22S		36.7%	2.5%	ε^2=0.294
	MscL-G22N		59.6%	4.5%	95% CIs = [-45.05, -8.64]
	MscS		47.3%	2.5%	
	CON	400 mW/cm^2	31.4%	2.5%	*P*<0.0001,***
	MscL-G22S		39.2%	2.5%	ε^2=0.380
	MscL-G22N		61.9%	3.5%	95% CIs = [-55.05, -16.87]
	MscS		47.9%	3.1%	
Theta	CON	100 mW/cm^2	25.7%	1.3%	*P*=0.0040,**
	MscL-G22S		25.5%	0.8%	ε^2=0.175
	MscL-G22N		21.1%	0.9%	95% CIs = [2.22, 37.51]
	MscS		22.6%	0.9%	
	CON	250 mW/cm^2	26.6%	1.3%	*P*=0.0009,***
	MscL-G22S		27.8%	1.6%	ε^2=0.222
	MscL-G22N		21.0%	1.0%	95% CIs = [2.63, 39.04]
	MscS		22.5%	1.1%	
	CON	400 mW/cm^2	27.3%	0.9%	*P*<0.0001,***
	MscL-G22S		25.6%	1.1%	ε^2=0.286
	MscL-G22N		20.7%	0.9%	95% CIs = [12.02, 51.08]
	MscS		22.2%	1.1%	

Supplementary Table S5 (continued table)

Band	Group	Intensity	mean	SEM	p-value
Alpha	CON	100 mW/cm^2	17.8%	1.6%	P=0.0005,***
	MscL-G22S		15.2%	1.1%	ε^2=0.249
	MscL-G22N		9.0%	1.6%	95% CIs = [9.39, 44.69]
	MscS		13.4%	0.8%	
	CON	250 mW/cm^2	15.9%	1.3%	P=0.0012,**
	MscL-G22S		15.2%	0.9%	ε^2=0.211
	MscL-G22N		9.1%	1.7%	95% CIs = [6.07, 42.48]
	MscS		13.1%	0.8%	
	CON	400 mW/cm^2	17.1%	1.2%	P<0.0001,***
	MscL-G22S		15.1%	1.0%	ε^2=0.351
	MscL-G22N		8.1%	1.3%	95% CIs = [14.71, 53.76]
	MscS		13.0%	1.1%	
Beta	CON	100 mW/cm^2	21.6%	2.4%	P=0.0006,***
	MscL-G22S		18.7%	1.6%	ε^2=0.243
	MscL-G22N		9.5%	1.9%	95% CIs = [7.48, 42.78]
	MscS		25.7%	1.3%	
	CON	250 mW/cm^2	19.8%	2.2%	P=0.0009,***
	MscL-G22S		17.9%	1.4%	ε^2=0.220
	MscL-G22N		9.2%	1.8%	95% CIs = [6.31, 42.72]
	MscS		15.5%	1.2%	
	CON	400 mW/cm^2	20.8%	1.6%	P<0.0001,***
	MscL-G22S		17.4%	1.2%	ε^2=0.346
	MscL-G22N		8.4%	1.5%	95% CIs = [14.97, 54.03]
	MscS		15.4%	1.4%	
Gamma	CON	100 mW/cm^2	2.6%	0.3%	P<0.0001,***
	MscL-G22S		2.6%	0.2%	ε^2=0.488
	MscL-G22N		1.1%	0.2%	95% CIs = [11.72, 47.01]

Supplementary Table S5 (continued table)

Band	Group	Intensity	mean	SEM	p-value
Gamma	MscS	100 mW/cm^2	1.6%	0.1%	
	CON	250 mW/cm^2	2.9%	0.4%	*P*<0.0001,***
	MscL-G22S		2.4%	0.2%	ε^2=0.340
	MscL-G22N		1.1%	0.2%	95% CIs = [11.45, 47.86]
	MscS		1.6%	0.1%	
	CON	400 mW/cm^2	3.4%	0.4%	*P*<0.0001,***
	MscL-G22S		2.7%	0.3%	ε^2=0.513
	MscL-G22N		1.0%	0.1%	95% CIs = [2.94, 44.62]
	MscS		1.6%	0.1%	

* P-values in Supplementary Table S5 are derived from Kruskal–Wallis tests followed by Dunn’s post-hoc pairwise comparisons performed within each frequency band and stimulus intensity.

This table presents detailed statistical comparisons of relative band-power distribution across conventional frequency bands (Delta: 0.5-4 Hz, Theta: 4-8 Hz, Alpha: 8-12 Hz, Beta: 12-30 Hz, Gamma: 30-100 Hz) in response to varying ultrasound intensities (100 mW/cm^2, 250 mW/cm^2, and 400 mW/cm^2). Significant group-specific effects were observed, particularly in the MscL-G22N group, which exhibited consistently elevated delta power and reduced power in higher frequency bands across all stimulation intensities. Statistical significance is indicated for relevant between-group comparisons, with significance levels denoted by asterisks (** $P < 0.01$, *** $P < 0.001$). Mean values and standard errors are provided for each experimental condition, demonstrating intensity-dependent modulation of neural oscillatory patterns specific to each mechanosensitive channel variant.

Expression level

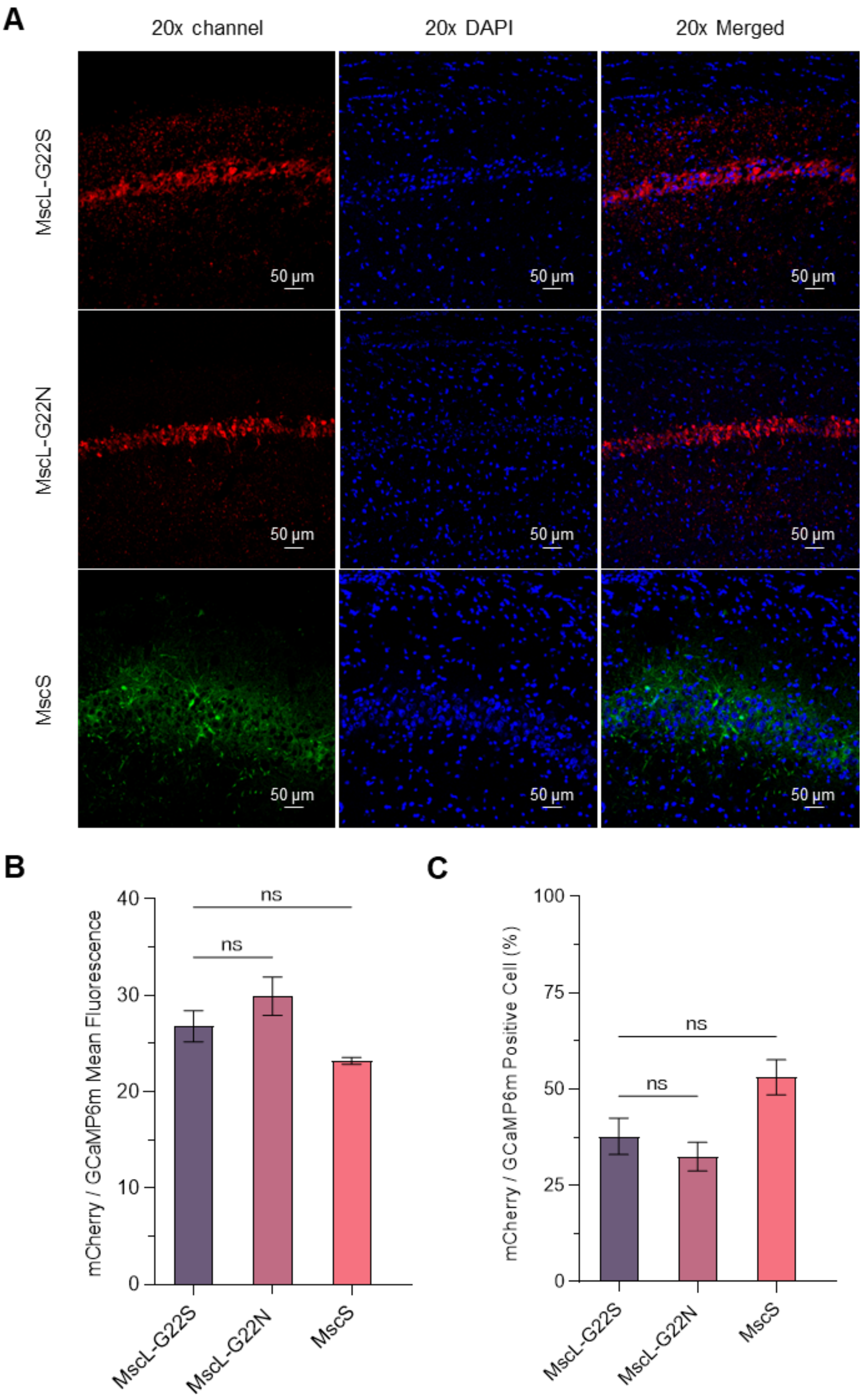

Supplementary Figure S6. Quantification of expression level of different mechanosensitive channels. (A) Representative fluorescence images of hippocampus CA1 region with reporter label (MscL-G22S and MscL-G22N, red; MscS, green; images at 20x magnification, scale bar: 50 μm). **(B)** Mean fluorescence intensity of mCherry/GCaMP6m in the hippocampus (MscL-G22S, n=6; MscL-G22N, n=6; MscS, n=6). **(C)** Percentage positive

cell of mCherry/GCaMP6m in the hippocampus (MscL-G22S, n=6; MscL-G22N, n=6; MscS, n=6). Kruskal-Wallis test with Dunn's post-hoc comparisons was used in **B** and **C**; the data are shown as mean ± SEM. ns, no significant. The fluorescence intensity comparisons between constructs are not directly comparable, and interpretations are limited to within-group analyses.

Behavior performance

OFT test results

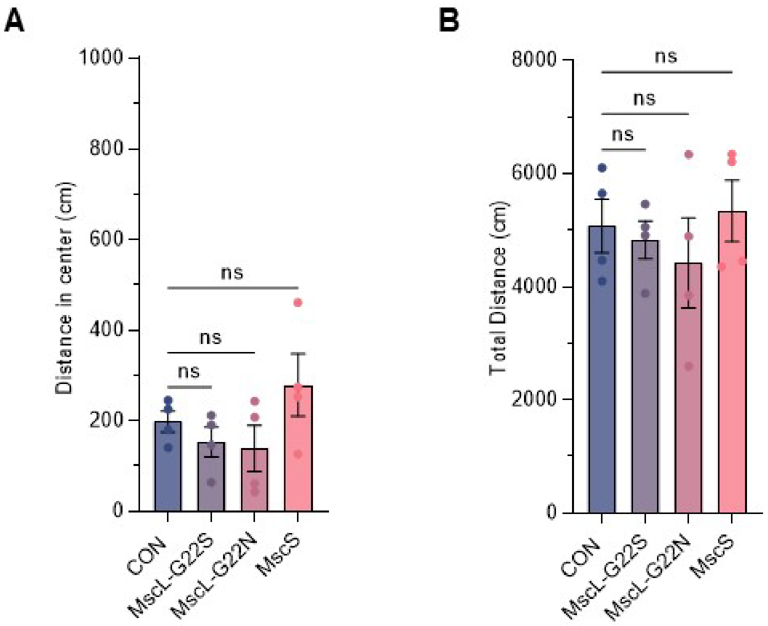

Supplementary Figure S7. Baseline behavior performance in OFT test. (A) Mean distance traveled in the center is (CON, n=4; MscL-G22S, n=4; MscL-G22N, n=4; MscS, n=4). **(B)** Total distance traveled in the entire area (CON, n=4; MscL-G22S, n=4; MscL-G22N, n=4; MscS, n=4). Kruskal-Wallis test followed by Dunn's post-hoc test was used for statistical analysis, and the data were shown as mean ± SEM. ns, no significant.

The open field test was conducted to evaluate the anxiety level of the rats. Four quantification indexes including distance in the center, entries count in the center, mean speed in the center, and total movement distance in the whole area were selected to analyze. Here display the summary results of distance in center (CON: 198.1 ± 23.36, MscL-G22S: 153.3 ± 32.87, MscL-G22N: 138.6 ± 50.64, MscS:278.6 ± 68.72; $P > 0.05$, Supplementary Figure S7A) and total distance (CON: 5080 ± 474, MscL-G22S: 4827 ± 335.4, MscL-G22N:4419 ± 793.8, MscS:5339 ± 542.5; $P > 0.05$, Supplementary Figure S7B). As the result demonstrated in the figures, there no

obvious anxiety behavior after the different MS channels expressed in the hippocampus.

EPM test results

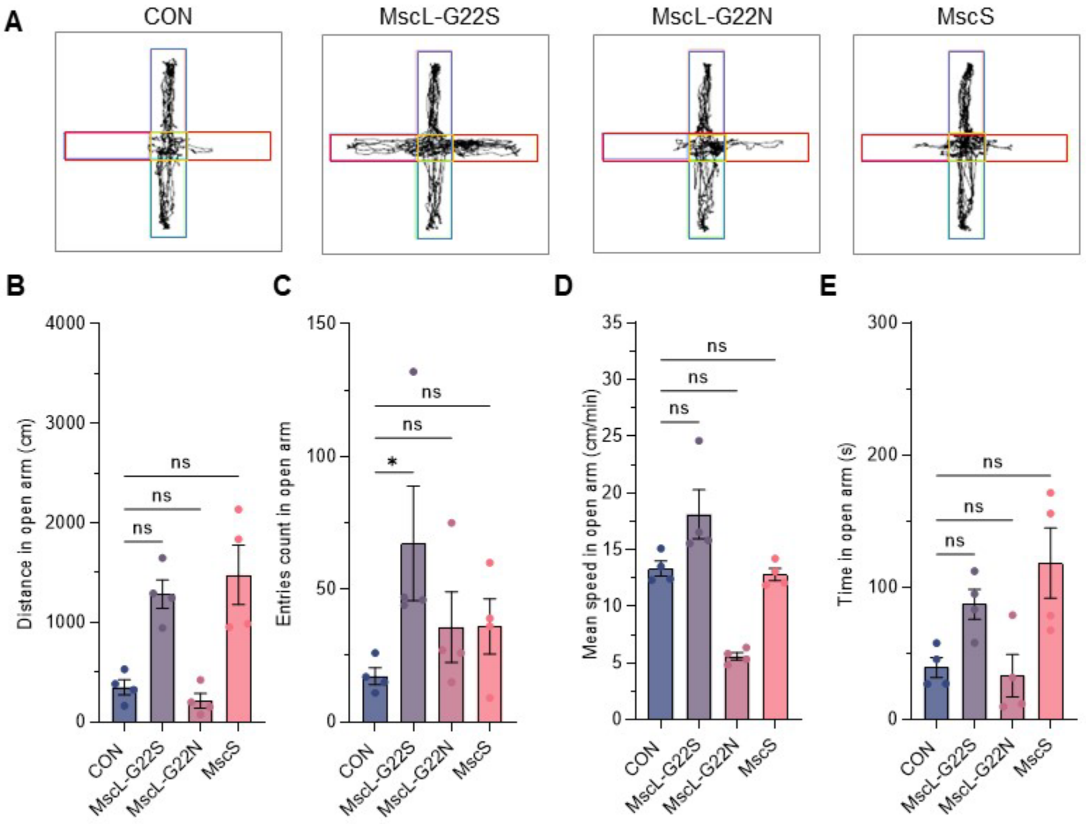

Supplementary Figure S8 Baseline behavior performance in the EPM test. (A) The Representative trajectories in EPM of different groups. **(B)** The total movement distance in open arm (CON, n=4; MscL-G22S, n=4; MscL-G22N, n=4; MscS, n=4). **(C)** The entries count in the open area (CON, n=4; MscL-G22S, n=4; MscL-G22N, n=4; MscS, n=4). **(D)** The mean speed in open arm (CON, n=4; MscL-G22S, n=4; MscL-G22N, n=4; MscS, n=4). **(E)** The exploration time in open arms (CON, n=4; MscL-G22S, n=4; MscL-G22N, n=4; MscS, n=4). Kruskal-Wallis test with Dunn's post-hoc comparisons was used in **B~E**, the data shown as mean ± SEM. ns, no significant.

To further evaluate the rats' anxiety level, the EPM test was selected. Similar results were found that no significant difference was found in the following four indicators between the CON group and other MS experimental groups (Supplementary Figure 8B-8E). The distance in open arm, mean speed in open arm, and time in open arm between groups showed no difference. Only in entries count in open arm displayed a significant difference between CON and MscL-G22S group (CON: 17.25 ± 3.17, MscL-G22S: 67.25 ± 21.59, $P = 0.0277$).

FST test results

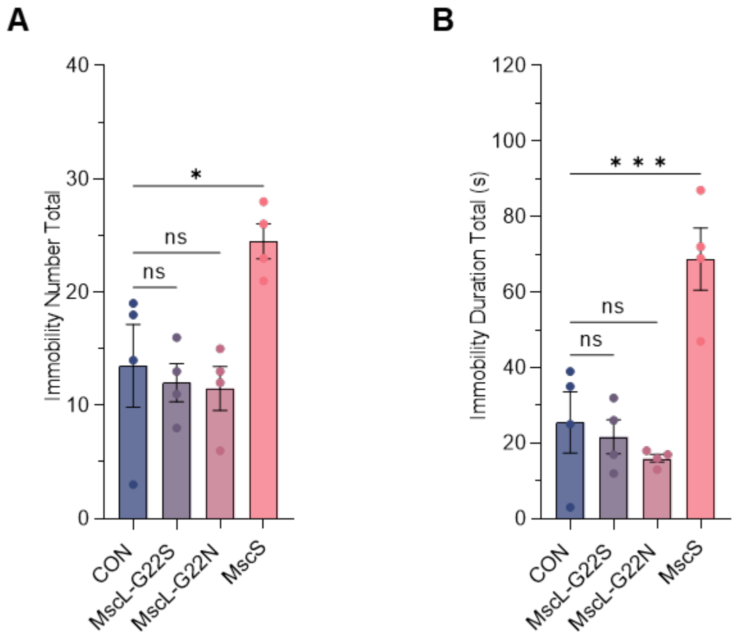

Supplementary Figure S9. Baseline behavior performance in the FST test. (A) Number of immobility episodes in the last 5 minutes (CON, n=4; MscL-G22S, n=4; MscL-G22N, n=4; MscS, n=4). **(B)** Total immobility duration in the last 5 minutes (CON, n=4; MscL-G22S, n=4; MscL-G22N, n=4; MscS, n=4). Kruskal-Wallis test with Dunn's post-hoc comparisons was used, the data are presented as the mean ± SEM. * $P < 0.05$, *** $P < 0.001$, ns, no significant.

To investigate whether the expression of mechanosensitive ion channels in the hippocampal region induces depressive-like behaviors in animals, the FST was employed for evaluation. The total immobility number (Supplementary Figure S9A) and total immobility duration (Supplementary Figure S9B) were selected to indicate the depressive-like level. Compared with CON, the MscL-G22S and MscL-G22N groups have no significant difference. However, the MscS group showed a significant increase in these two indices (total immobility number: CON vs. MscS, $P = 0.0169$; total immobility duration: CON vs. MscS, $P = 0.001$).

Y-maze test results

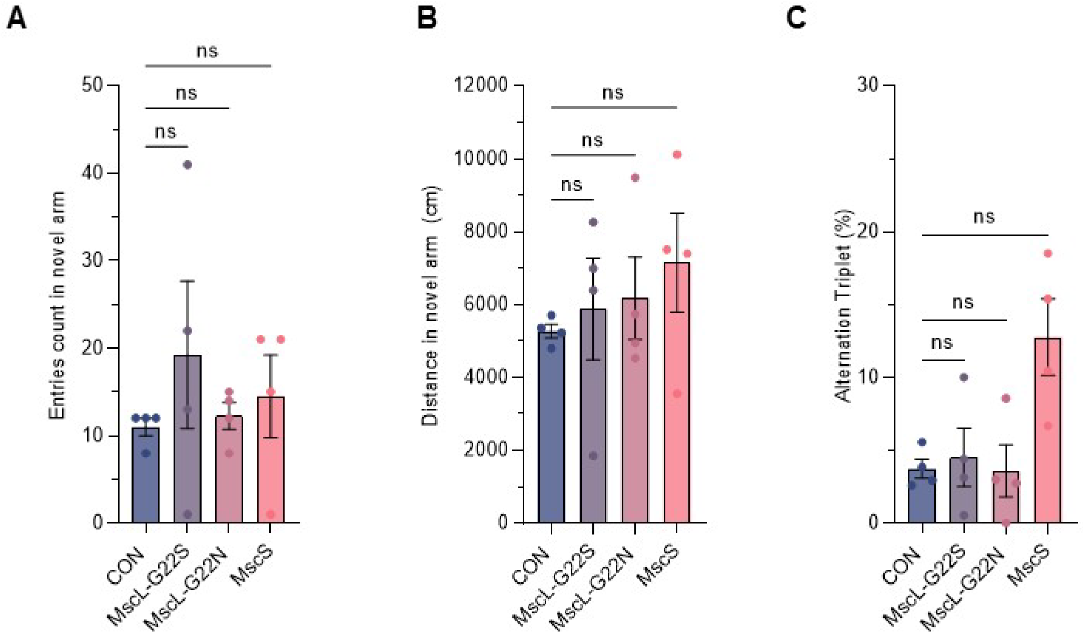

Supplementary Figure S10. Baseline behavior performance in the Y-maze test. (A) The total entries count in the novel arm for the Y-maze novel arm test (CON, n=4; MscL-G22S, n=4; MscL-G22N, n=4; MscS, n=4). **(B)** The total movement distance Y-maze novel arm test (CON, n=4; MscL-G22S, n=4; MscL-G22N, n=4; MscS, n=4). **(C)** The alternation triplet ratio for the Y-maze spontaneous alternation test (CON, n=4; MscL-G22S, n=4; MscL-G22N, n=4; MscS, n=4). Kruskal-Wallis test with Dunn's post-hoc comparisons was used, the data are presented as the mean ± SEM. ns, no significant.

The short-term spatial cognition of the animals was measured by the Y-maze novel arm and Y-maze spontaneous alternation tests. The entries in the novel arm (Supplementary Figure S10A) and distance in the novel arm (Supplementary Figure S10B) displayed no significant difference between CON and other MS experimental groups. In addition, in the Y-maze spontaneous alternation test, no significant differences in alternation triplet were observed among the different MS channel groups (Supplementary Figure S10C). All these results indicate that the expression of mechanosensitive ion channels did not affect the animals' short-term spatial learning and memory abilities.
